# The effect of *S*-alkylation on organocatalytic enamine activation through imidazolidine-4-thiones†

**DOI:** 10.1039/d3cc01912h

**Published:** 2023-06-09

**Authors:** Magenta J. Hensinger, Anna C. Closs, Oliver Trapp, Armin R. Ofial

**Affiliations:** a Department Chemie, Ludwig-Maximilians-Universität München Butenandtstr. 5-13 München 81377 Germany oliver.trapp@cup.uni-muenchen.de ofial@lmu.de; b Max-Planck-Institute for Astronomy Königstuhl 17 Heidelberg 69117 Germany

## Abstract

Imidazolidine-4-thiones have been suggested as potential prebiotic organocatalysts for light-driven α-alkylations of aldehydes by bromoacetonitrile. However, imidazolidine-4-thiones react with bromoacetonitrile to give *S*-cyanomethylated dihydroimidazoles. Kinetic studies show that enamines derived from these cyclic secondary amines and aldehydes are more nucleophilic than enamines derived from aldehydes and MacMillan organocatalysts.

## Abstract 2

Enamine intermediates in the organocatalytic α-cyanomethylation of enolisable aldehydes were identified and their reactivities on the Mayr nucleophilicity scale determined.

## Graphical abstract

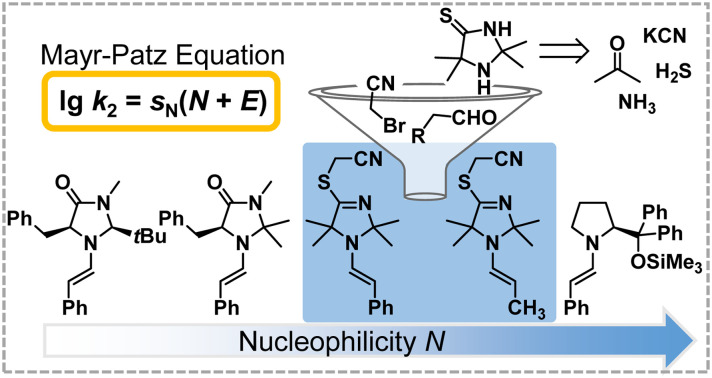

Five-membered cyclic secondary amine catalysts have proven their outstanding capacities in organocatalytic organic synthesis over the past two decades. For example, MacMillan's imidazolidine-4-ones or prolinol-based organocatalysts activate carbonyl compounds for subsequent transformations by enamine or iminium ion formation.^1^

Intermolecular α-cyanoalkylation of enolisable aldehydes was considered difficult because of the weak electrophilicity of alkyl halides, which hindered efficient reactions with nucleophilic enol derivatives. This issue has been overcome by MacMillan (Fig. 1a), who elegantly combined metal-based photoredox catalysis with enamine activation to α-alkylate aldehydes by bromoacetonitrile (BAN).^2^ The nitrile group in the product aldehydes beneficially introduced a wide range of options for further synthetic conversions.

**Fig. 1:**
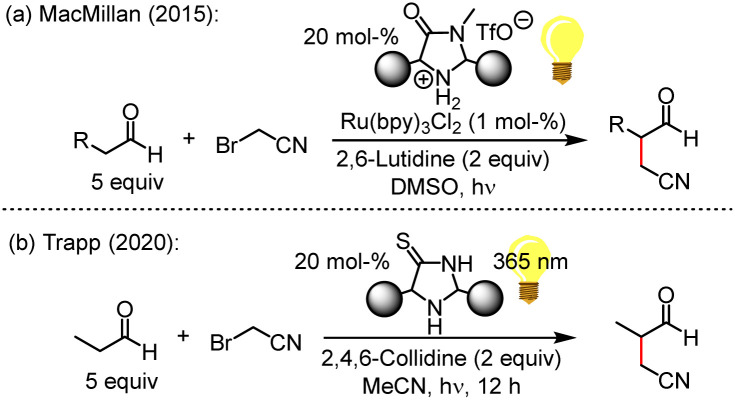
α-Alkylation of aldehydes using imidazolidine-4-(thi)one-based organocatalysts.

Light-induced radical reactions of elementary bromine and acetonitrile^3*a*,*b*^ may have led to BAN formation on the Early Earth. Motivated by this aspect, the Trapp group suggested a light-driven α-cyanomethylation of propanal by BAN that used imidazolidine-4-thiones (ITOs) as catalysts (Fig. 1b).^4^ ITOs are the thiolactam analogues^5^ of imidazolidine-4-ones (MacMillan catalysts), which feature a lactam moiety. Remarkably, ITOs assemble modularly from simple building blocks, that is, KCN, H_2_S, NH_3_, ketones or aldehydes.^6^ Thus, also the existence of ITOs under prebiotic conditions on the Early Earth seems likely,^7^ and ITO-catalysed α-alkylations of aldehydes by BAN may have led to the non-enzymatic formation of more complex, functionalized carbonyl compounds. The reagents and conditions used in Fig. 1b establish an organocatalytic version^8^ of the aldehyde α-alkylation,^9^ which is compatible with plausible prebiotic conditions.^3,4,7^

Most interestingly, ITO formation is reversible, and dynamic exchange of the carbonyl building blocks enables ITOs to adapt to environmental conditions. This process may provide a mechanism for molecular evolution toward structurally modified 2^nd^ generation ITOs.^7^

Yet, insight in the role of ITOs in the reaction shown in Fig. 1b has remained vague so far. On the one hand, it is known that the thiolactam group of ITOs is easily *S*-alkylated by alkyl halides or Michael acceptors to give 4-(alkylthio)-2,5-dihydro-1*H*-imidazoles (TIMs).^10,11^ On the other hand, it is widely accepted that the α-cyanomethylation of enolisable aldehydes requires enamine activation as a crucial step in the catalytic cycle. Given that enamine formation is slow and reversible but the competing *S*-alkylation is fast and irreversible, we suspected that ITOs are only the precursors of the effective species in the catalytic reactions shown in Fig. 1b.

We, therefore, set out to trace ITO-derived intermediates in the α-cyanomethylation of aldehydes by BAN. Potential enamine intermediates in the catalytic cycle were independently prepared and their nucleophilicity was determined by using Mayr's benzhydrylium methodology^12^ and the Mayr–Patz eqn (1), which is a linear free energy relationship.

1 lg *k*_2_(20 °C) = *s*_N_(*N* + *E*)

In eqn (1), *E* represents the electrophilicity of the (reference) electrophile, while the nucleophilicity is characterised by two solvent-dependent parameters *N* and *s*_N_.^13^Eqn (1) has already been used to successfully quantify the nucleophilicities of prolinol-based and MacMillan organocatalysts^14^ as well as of enamines derived thereof.^15^ As a consequence, the reactivity of the enamines studied in this work can straightforwardly be compared with relevant species that occur in many, more commonly used, enamine-activated reactions.^13*e*,15^ Hence, a quantitative fundament for the understanding of both prebiotic relevance of ITO catalysts and their scope in organocatalytic reactions is provided in this work.

The previously discovered light-driven alkylation of propanal by BAN was re-investigated in the dark under otherwise identical conditions except for the use of 2,6-lutidine as the sterically hindered Brønsted base instead of 2,4,6-collidine (Fig. 2).

**Fig. 2:**
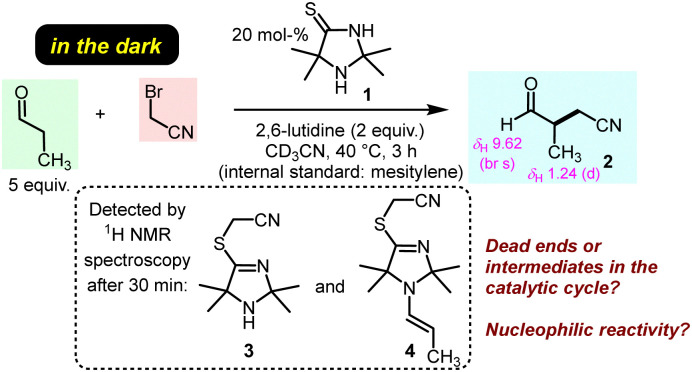
ITO-promoted α-cyanomethylation of propanal with BAN in the dark.

The 2,2,5,5-tetramethylated ITO 1 was selected because it had proven superior efficiency in the analogous light-driven α-alkylation compared to ITOs with other alkylation patterns.^4^ To simulate the temperature under intense irradiation, the reaction mixture was mildly heated at 40 °C. Interestingly, ITO 1 catalysed the α-alkylation of propanal with BAN without the need of excitation by light as evidenced by the occurrence of the characteristic resonances for the formyl and the methyl protons in 3-methyl-4-oxobutanenitrile (2) in the ^1^H NMR spectrum of the reaction mixture (Fig. 2 and ESI,† Fig. S1). Thus, light is not necessary to promote the α-alkylation of aliphatic aldehydes by BAN with ITO 1 as the organocatalyst.

Time-resolved ^1^H NMR spectroscopy enabled tracing of the ITO-derived intermediates over the course of the reaction. We observed that resonances of the initial ITO catalyst 1 decayed almost completely within 2 h. Meanwhile, further resonances were detected in the reaction mixture which were assigned to the *S*-alkylated ITO, that is TIM 3, and the TIM-derived enamine 4 (Fig. 2 and ESI,† Fig. S1).

We next investigated whether the TIM-derived enamine 4 is a dead end of the reaction or whether it is the crucial reactive intermediate that undergoes the C–C bond formation with BAN in ITO-driven α-alkylations of aliphatic aldehydes under the conditions of Fig. 2. We, therefore, prepared samples of the enamine 4 by irradiating a mixture of TIM 3, propanal and 2,6-lutidine in dry acetonitrile with light (420 nm). Additionally, we investigated the enamine 5 derived from 3 and phenylacetaldehyde. Enamine 5 allows one to assess the activating effect of TIM 3 by comparing it with a wide range of enamines derived from the same aldehyde and various types of cyclic secondary amines. Enamines derived from established organocatalysts have already been reported on the Mayr nucleophilicity scale and characterised by *N* and *s*_N_.^13*e*,15^

As indicated by the ^3^*J* coupling constants of the olefinic protons, the carbon–carbon double bonds in 4 and 5 are both in (*E*)-configuration. In the crystal structure, the phenylacetaldehyde-derived 5 adopts an *s-cis* conformation (Fig. 3). Given that both positions 2 and 5 at the heterocycles of 4 and 5 carry two methyl groups, we neglected to discuss the conformational orientation at the N–C(α) bond of the enamines in the subsequent reactivity studies.

**Fig. 3:**
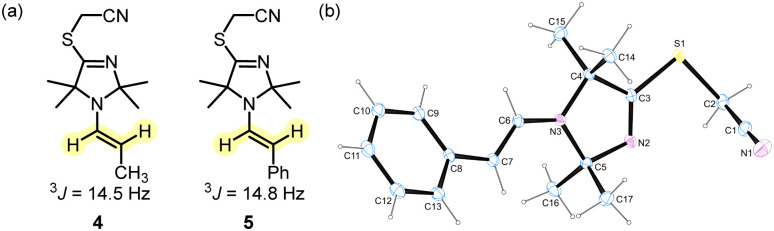
(a) Selected ^3^*J* coupling constants in enamines 4 and 5 (400 MHz, CD_3_CN). (b) X-Ray structure of enamine 5 (CCDC 2254388).†

Under irradiation with UV light (365 nm, in MeCN with 2,6-lutidine as a base), the TIM-derived enamine 4 reacted cleanly with BAN to give the α-alkylated enamine product 6 (structure shown in Fig. 4, details are given in the ESI†).

**Fig. 4:**
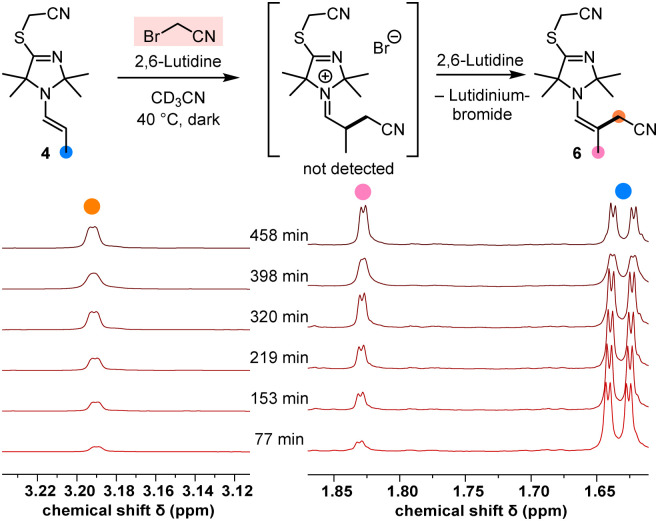
^1^H NMR spectroscopic monitoring of the α-alkylation of the enamine 4 by BAN in CD_3_CN at 40 °C (2.7 equiv. of 2,6-lutidine).

Moreover, NMR spectroscopic monitoring demonstrated that BAN also alkylated the enamine 4 in the dark at 40 °C (Fig. 4), which underlined the reactive capacity of the nucleophilic TIM-derived enamine intermediate 4 in a polar catalytic cycle.

Since we started from the preformed enamine 4, the intermediate iminium bromide was not hydrolysed (as observed in the catalytic reaction, Fig. 2) but rather deprotonated by 2,6-lutidine to generate the α-cyanomethylated enamine 6. Owing to the fact that S_N_2 electrophiles cannot straightforwardly be used as references for integrating nucleophiles in Mayr's comprehensive reactivity scales, we decided to use the benzhydrylium methodology^12^ to calibrate the nucleophilicities of the TIM-derived enamines 4 and 5.

Benzhydrylium tetrafluoroborates 7 (Fig. 5) are widely used reference electrophiles with solvent-independent and reliably determined electrophilicity parameters *E*.^12,13*b*,*f*^ Reactions of 7 with the enamines 4 and 5 and subsequent aqueous workup furnished the corresponding α-benzhydrylated aldehydes 9 in unoptimised yields of 63 to 74% (ESI†).

**Fig. 5:**
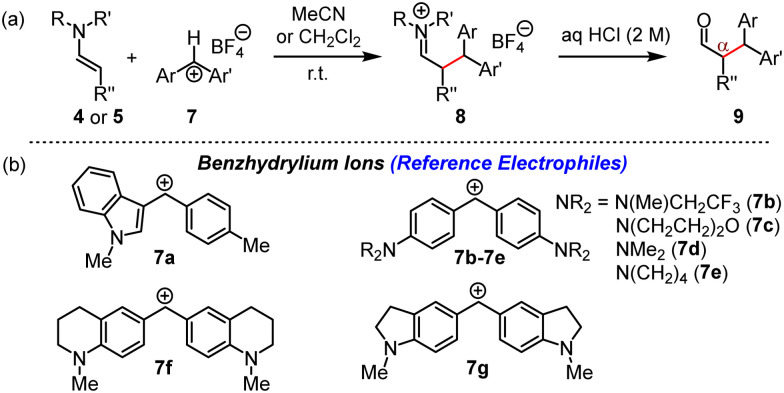
(a) Reactions of 4 or 5 with 7 furnished iminium salts 8, which were hydrolysed to α-alkylated aldehydes 9. (b) Structures of reference electrophiles 7a–7g (counterion: tetrafluoroborate).

The kinetics of the reactions of 4 and 5 with 7a–7g in acetonitrile at 20 °C were monitored photometrically by following the consumption of 7 (stopped-flow method, 434 nm ≤ *λ*_max_ ≤ 620 nm, 4 or 5 in >10-fold excess). First-order rate constants *k*_obs_ (s^−1^) were derived by least squares fitting of the function *A*_t_ = *A*_0_ exp(−*k*_obs_*t*) + *C* to the absorbance decays of 7. The second-order rate constants *k*_2_ (M^−1^ s^−1^) compiled in Table 1 were then obtained as the slopes of the linear correlations of *k*_obs_ with the concentration of the enamines 4 or 5. Details of the individual kinetic measurements are given in the ESI.† Analogous kinetic studies on the nucleophilicity of 5 in dichloromethane showed that the reactivity of 5 did not differ significantly from that in acetonitrile, in accord with previously reported marginal solvent effects on reactions of enamines with cationic electrophiles.^16^

**Table 1:** Second-order rate constants *k*_2_ for the reactions of the enamines 4 or 5 with the benzhydrylium tetrafluoroborates 7 and Mayr nucleophilicity parameters for 4 and 5 (acetonitrile, 20 °C)

Enamine	Electrophile	Mayr *E*^a^	*k*_2_ (M^−1^ s^−1^)	*N*, *s*_N_
4	7d	–7.02	7.06 × 10^1^	9.25, 0.84
	7e	–7.69	2.14 × 10^1^	
	7f	–8.22	7.74	
	7g	–8.76	2.42	
5	7a	–2.19	6.92 × 10^4^	7.74, 0.87
	7b	–3.85	1.96 × 10^3^	
			(1.75 × 10^3^)^b^	
	7c	–5.53	8.84 × 10^1^	
			(5.49 × 10^1^)^b^	

a Electrophilicity parameters *E* (as defined in eqn (1) and reported in ref. 13).

b In dichloromethane. With further data in ESI, *N*(*s*_N_) = 7.06 (1.11) was calculated for 5 in CH_2_Cl_2_.

Fig. S5 (ESI†) illustrates that the second-order rate constants (lg *k*_2_) for the reactions of 7 with the enamines 4 and 5 correlate linearly with the reported Mayr electrophilicities *E* of the reference electrophiles 7. By using eqn (1), the electrophilicities *E*(7), and the experimentally determined second-order rate constants *k*_2_, we calculated the nucleophilicity parameters *N* (and *s*_N_) of the enamines 4 and 5, which are listed in Table 1.

The now available Mayr reactivity descriptors *N* for the enamines 4 and 5 facilitate a comparison in the context of previously studied enamines which are key species in organocatalytic reactions.‡‡Given that the *s*_N_ values of the enamines depicted in Fig. 6 are in a narrow range (0.84 to 1.14), we can directly compare their nucleophilic reactivity by only considering the nucleophilicity parameter *N*. The TIM-derived enamines 4 and 5 are considerably more reactive than structurally analogous enamines derived from MacMillan catalysts (Fig. 6). However, enamines derived from the diphenylprolinol trimethylsilyl ether (Hayashi–Jørgensen catalyst) exceed 4 and 5 in nucleophilicity by one to three orders of magnitude.^13*e*,15,17*a*^

**Fig. 6:**
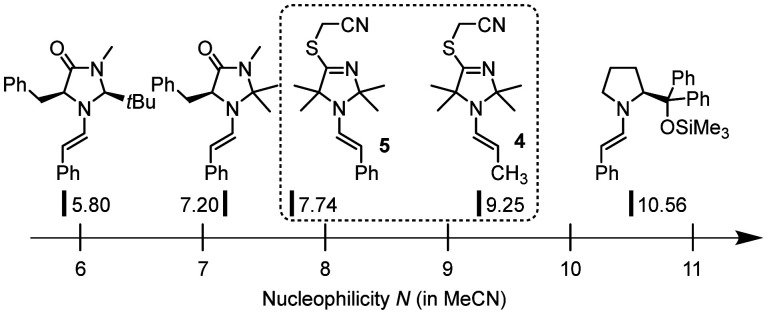
Comparison of nucleophilic reactivities of enamines 4 and 5 derived from TIM 3 with those of structural analogues derived from MacMillan's organocatalysts or the diphenylprolinol trimethylsilyl ether (with *N* parameters reported in ref. 13*e* and 15).

In conclusion, we suggest TIMs as a new generation of organocatalytically active cyclic secondary amines, which are generated through *S*-alkylation of ITOs by BAN. Instead of the previously proposed ITOs,^4^ TIMs are the active catalysts in the α-alkylation of aldehydes (Fig. 7a). Without activation by light, the catalytic cycle starts with enamine activation of aldehydes by TIM, which generates a sufficiently nucleophilic enamine to undergo C–C bond-forming reactions with electrophiles, such as BAN. Previously studied ITO-catalysed reactions were carried out under conditions that could not benefit from TIM formation or the activating effect of *S*-alkylation on the TIM-derived enamine reactivity.^5^ Thus, the *S*-alkylation step opens the gate to a novel TIM generation of organocatalysts for organic synthesis.

**Fig. 7:**
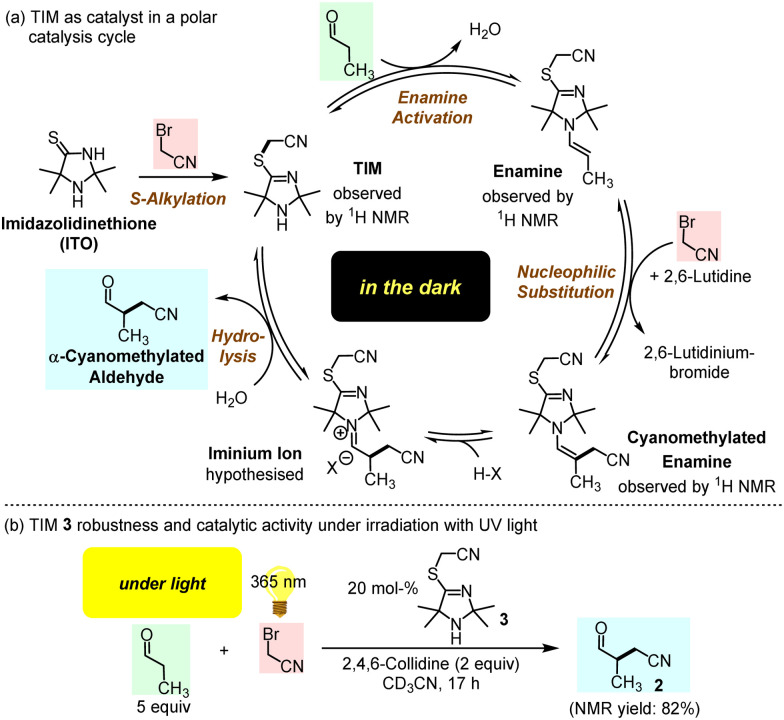
(a) Proposed catalytic cycle for the α-alkylation of propanal in the dark. (b) Performance of TIM 3 in a photon-driven α-cyanomethylation of propanal by BAN (under identical conditions, the slower polar reaction in the dark furnished 2 in 6% yield after 22 h at r.t.).

Furthermore, *S*-cyanomethylation of ITOs to give TIMs may have been a beneficial step in molecular evolution under Early Earth conditions for the following reasons: (1) TIMs can assemble from prebiotically likely small molecules (KCN, H_2_S, NH_3_, short-chain carbonyl compounds, and BAN). (2) In the dark TIMs facilitate through enamine activation α-alkylations of aldehydes by electrophiles. (3) Once formed organic molecules should not degrade rapidly under the harsh conditions on the Early Earth. Remarkably, preliminary studies have shown that TIM 3 does not degrade when exposed to UV light (365 nm) but performs well as a catalyst (Fig. 7b and ESI†).^9,17^

We thank Dr Peter Mayer (Dept. Chemie, LMU München) for the X-ray structure determination and Nathalie Hampel for preparing the reference electrophiles 7. This project has received funding from the European Union's Horizon 2020 research and innovation programme under the Marie Skłodowska-Curie grant agreement No. 101024710 “SImCat” (MSCA-IF-2020 to M. J. H.). We acknowledge financial support from the VolkswagenStiftung, “Initiating Molecular Life” (O.T.).

## Conflicts of interest

There are no conflicts to declare.

## Supplementary Material

CC-059-D3CC01912H-s001

CC-059-D3CC01912H-s002
