# Supplementary material for: The effect of S-alkylation on organocatalytic enamine activation through imidazolidine-4-thiones
Source: Chem Commun (Camb). 2023 Jun 9;59(52):8091–4. doi: 10.1039/d3cc01912h (PMC10297834; doi:10.1039/d3cc01912h)
Supplement: CC-059-D3CC01912H-s001 [file CC-059-D3CC01912H-s001.pdf]

Electronic Supplementary Material (ESI) for Chemical Communications.
This journal is © The Royal Society of Chemistry 2023

Electronic Supplementary Information (ESI)

The effect of S-alkylation on organocatalytic enamine activation through imidazolidine-4-thiones

Magenta J. Hensinger,[a] Anna C. Closs[a,b], Oliver Trapp*[a,b] and Armin R. Ofial*[a]

[a] Department Chemie, Ludwig-Maximilians-Universität München

Butenandtstr. 5-13, 81377 München, Germany.

E-mail: oliver.trapp@cup.uni-muenchen.de, ofial@lmu.de

[b] Max-Planck-Institute for Astronomy, Königstuhl 17, 69117 Heidelberg, Germany.

Table of Contents

1. General S3

2. Preliminary studies of the α-alkylation of propanal S5

3. Synthesis S8

4. Crystallographic data for enamine **5** S11

5. Reactions of enamines **4** or **5** with benzhydrylium tetrafluoroborates **7** S12

6. Evidence corroborating the formation of enamine **6** and NMR reaction monitoring S16

6.1 Formation of enamine **6** under photoirradiation S16

6.2 Formation of enamine **6** in the dark S21

7. (TIM **3**)-catalysed reaction (under irradiation with 365 nm light) S24

8. (TIM **3**)-catalysed reaction (in the dark) S29

9. Kinetic experiments S31

9.1 Kinetics of the propanal-derived enamine **4** in acetonitrile S31

9.2 Kinetics of the cinnamaldehyde-derived enamine **5** in acetonitrile S34

9.3 Correlations of lg k_2 for reactions of **7** with **4** and **5** in acetonitrile with the electrophilicities *E* of **7** S35

9.4 Kinetics of the cinnamaldehyde-derived enamine **5** in dichloromethane S36

10. Copies of ^{1}H NMR, ^{13}C NMR, and IR spectra S39

11. References S58

1. General

Chemicals. Chemicals used in the synthesis were purchased from commercial sources (Acros Organics, Sigma-Aldrich, Alfa Aesar by Thermo Fischer Scientific, VWR International GmbH (Avantor), and TCI Europe) and used without purification unless otherwise indicated. Tetrahydrofuran was dried using sodium metal and distilled prior to use. Commercially available acetonitrile (99% extra dry over molecular sieves, Acros Organics), toluene (≥99.7% pure, Sigma-Aldrich), or dichloromethane (HPLC grade, VWR) used in synthetic procedures were used without purification. Molecular sieves (4 Å) were heated to 350 °C under vacuum for 6 h and then stored in a desiccator. All reactions were performed using dried glassware (dried using a heat gun under vacuum) under an atmosphere of nitrogen or argon except when using aqueous reagents.

Reactions were monitored using thin layer chromatography with either silica gel 60 aluminum backed plates with F-254 fluorescence indicator or neutral aluminium oxide 60 aluminium backed plates with F-254 fluorescence indicator (both from Merck, Darmstadt). Pentane was distilled prior to use for column chromatography. Triethylamine was used without purification. Flash column chromatography was performed on either silica gel 60 (0.040-0.063 nm) purchased from Merck, Darmstadt, or neutral aluminium oxide from Sigma Aldrich.

2,2,5,5-Tetramethyl-imidazolidin-4-thione (**1**) was prepared as reported in ref.[S1] Enamines **4** and **5** decompose over time in the presence of moisture and were, therefore, stored in a glovebox freezer (at < –30 °C) under a dry argon atmosphere. Benzhydrylium tetrafluoroborates **7a**–**7g** (reference electrophiles) were synthesized as described in ref.[S2]

Analytics. ^{1}H and $^{13}C\{^{1}H\}$ NMR spectra were recorded at 400, 600, or 800 MHz and at 101, 151, or 201 MHz, respectively. The chemical shifts *(*δ) for ^{1}H and ^{13}C nuclei are given in ppm relative to the signals of the solvents ($CDCl_3$: δ_H = 7.26 ppm and δ_C = 77.2 ppm; CD_3CN; δ_H = 1.94 ppm and δ_C = 118.3 ppm).[S3] Coupling constants are given in Hz, and the assignments of NMR signals are based on additional 2D-NMR experiments (gHSQC, gHMBC, COSY, and NOESY). Multiplicities are abbreviated as follows: s = singlet, d = doublet, t = triplet, q = quartet, h = hextet, m = multiplet, br

s = broad singlet. High-resolution mass spectra (HRMS) were obtained by using a Thermo Finnigan MAT 95 instrument (EI) or a Thermo Finnigan LTQ FT (ESI). IR spectra of neat compounds were recorded on an FTIR Spectrometer SPECTRUM BX II (Perkin Elmer) with ATR probe (diamond). Melting points were measured using a Büchi melting-point M-560 device and are not corrected. X-ray data was measured on a Bruker D8 Venture TXS system equipped with a multilayer mirror monochromator and a Mo Kα rotating anode X-ray tube (λ = 0.71073 Å).

Kinetics. The kinetics of the reactions of the enamines **4** and **5** with the electrophiles **7a**–**7g** were followed by UV/Vis spectroscopy by using an Applied Photophysics SX.20 stopped-flow spectrophotometer (10 mm light path). A constant temperature (20.0 ± 0.2 °C) was maintained through the use of a circulating bath cryostat. All solutions were prepared under an atmosphere of argon or nitrogen with HPLC grade acetonitrile (VWR) flushed with nitrogen or freshly distilled dichloromethane. The kinetic stopped-flow measurements were initiated by mixing equal volumes of acetonitrile (or dichloromethane) solutions of the nucleophiles and electrophiles. Nucleophile concentrations were at least ten times higher than electrophile concentrations to achieve pseudo-first order kinetics.

A conventional UV/Vis spectroscopic work station (J&M TIDAS diode array spectrometer with Hellma quartz insertion probe, circulating bath cryostat) was used to follow the kinetics of the slow reaction of **4** with **7g** at 20 °C in acetonitrile.

The first-order rate constants k_{obs} (s^{-1}) were obtained from the decay of the absorbance at or close to the absorption maximum of the coloured reference electrophiles by least squares fitting of the equation $A_t = A_0 \exp(-k_{obs}t) + C$ to the exponential absorption decay curve. Plots of k_{obs} (s^{-1}) versus the nucleophile concentration gave the second-order rate constants k_2 (M^{-1} s^{-1}) as slopes of the linear correlations.

2. Preliminary studies of the α-alkylation of propanal

2,2,5,5-Tetramethylimidazolidine-4-thione **1** (0.12 mmol, 0.2 equiv) was dissolved in CD_3CN (0.3 mL). Bromoacetonitrile (42 µL, 0.60 mmol, 1.0 equiv), 2,6-lutidine (140 µL, 1.20 mmol, 2.0 equiv), and propanal (215 µL, 3.0 mmol, 5.0 equiv) were added, and the mixture was stirred under the respective conditions as specified in Table S1. Samples were taken after 3 h and directly analysed by NMR spectrometry. The yield was determined by comparing the integral of the product aldehyde signal with that of the aromatic signal of mesitylene (15 µL, 0.108 mmol) as internal standard (see Figure S1).

Table S1. Reaction conditions for the organocatalytic α-cyanomethylation of propanal.

Entry	Conditions	Yield of aldehyde **2** (%)[a]
1	Irradiation with light (365 nm)	76
2	Irradiation with light (365 nm) at 0 °C	75
3	40 °C	25
4	40 °C, dark glass	24
5	40 °C, aluminium foil	25
6	room temperature	10
7	Without catalyst **1** at 40 °C	No reaction

[a] Yields refer to reaction times of 3 h and were determined by comparison to mesitylene as internal integration standard.

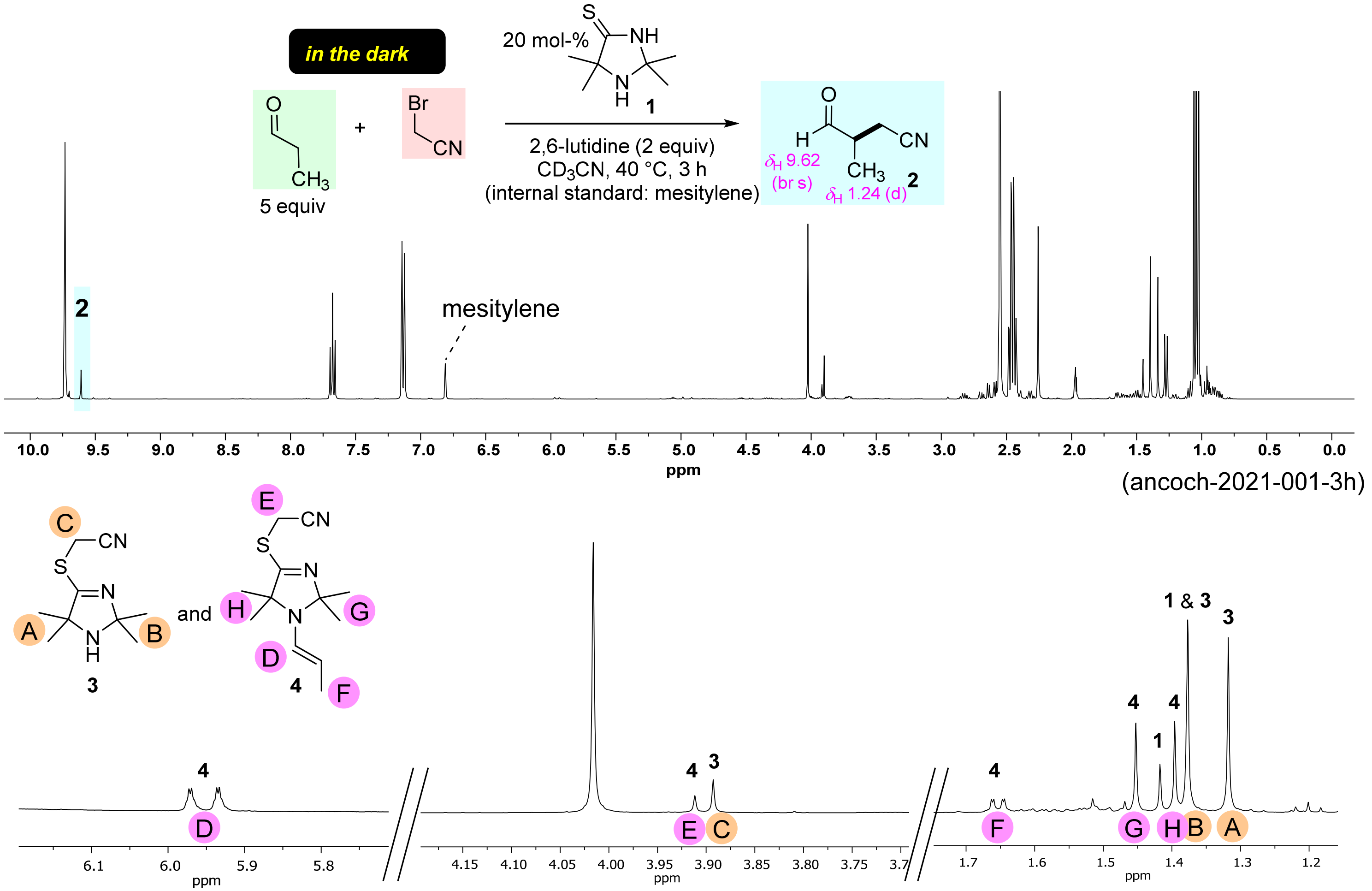

Figure S1. Exemplary ^{1}H NMR spectra (400 MHz, CD_3CN) of the α-cyanomethylation of propanal with bromoacetonitrile catalysed by 2,2,5,5-tetramethylimidazolidine-4-thione (**1**). Top: Full ^{1}H NMR spectrum after 3 h to illustrate the determination of the yield. Bottom: Merged excerpts of the ^{1}H-NMR spectrum after 30 min to illustrate the corresponding resonances of all imidazolidine-4-thione derivatives, that is, **1**, **3**, and **4**.

The NMR resonances for **3** were assigned by comparison with the 1D and 2D NMR spectroscopic data of an independently prepared sample of **3** (see Section 3). The formation of **3** was also observed when 2,2,5,5-tetramethylimidazolidine-4-thione (**1**) was mixed with bromoacetonitrile and 2,6-lutidine in acetonitrile without the addition of propanal.

The enamine **4** was only detected when 2,2,5,5-tetramethylimidazolidine-4-thione (**1**) reacted with a mixture of propanal and bromoacetonitrile or when preformed **3** reacted with propanal. The structure assignment was corroborated by comparison with the 1D and 2D NMR spectra of an independently prepared sample (see Section 3).

3. Synthesis

2-((2,2,5,5-Tetramethyl-2,5-dihydro-1*H*-imidazol-4-yl)thio)acetonitrile (3) (MJH-I-75)

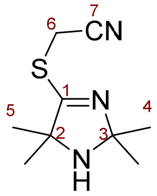

3

To a solution of NaH (60% dispersion in mineral oil, 2.1 equiv.) in freshly distilled THF (4 mL/mmol NaH) was added a solution of 2,2,5,5-tetramethylimidazolidine-4-thione **1** (1.56 g, 9.86 mmol) in freshly distilled THF (9 mL/mmol) dropwise at room temperature. After the complete addition, the reaction was left to stir for 10 min, and bromoacetonitrile (1.4 mL, 20.1 mmol) was added dropwise to the solution. The reaction mixture was left to stir until the disappearance of starting material was detected by TLC. The solvent was evaporated in the vacuum, and the solid residues were dissolved in H_2O (50 mL). The aqueous phase was extracted with diethyl ether (3 x 25 mL). The combined organic layers were washed with brine (30 mL), dried over $MgSO_4$, and the volatiles removed in the vacuum. The brown and solid crude product was purified by silica gel flash chromatography (silica gel, CH_2Cl_2/Et_2O 1:1) to furnish **3** (1.42 g, yield: 73%) as yellow needles, m.p. 72.2 °C; R_f = 0.26 (silica gel, EtOAc).

^{1}H NMR (600 MHz, $CDCl_3$): δ 3.86 (s, 2 H, 6-H), 1.88 (br. s, 1 H, NH), 1.42 (s, 6 H, 4-H), 1.35 (s, 6 H, 5-H). **^{13}C{^{1}H} NMR** (151 MHz, $CDCl_3$): δ 171.0 (C, C-1), 116.3 (C, C-7), 89.6 (C, C-3), 70.3 (C, C-2), 30.7 (CH_3, C-4), 28.7 (CH_3, C-5), 16.6 (CH_2, C-6). **IR** (ATR, neat): 3309, 2972, 2925, 2244, 1601, 1456, 1439, 1376, 1366, 1214, 1162, 1142, 1031, 966, 824, 777 cm^{-1}. **HRMS** (EI): *m/z* calcd for $C_9H_{15}N_3S^{\bullet+}$ [$M^{\bullet+}$]: 197.0981; found: 197.0988.

(*E*)-2-((2,2,5,5-Tetramethyl-1-(prop-1-en-1-yl)-2,5-dihydro-1*H*-imidazol-4-yl)thio)acetonitrile (**4**) (MJH-I-116//MJH-I-124)

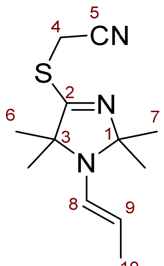

4

A solution of the secondary amine **3** (124 mg, 0.628 mmol), propanal (220 µL, 3.07 mmol), 2,6-lutidine (590 µL, 5.06 mmol), dry acetonitrile (0.70 ml/mmol), and 4 Å molecular sieves was stirred and irradiated for 5 h with a Roithner LaserTechnik-H2A1-H420 emitter (420 nm, light source placed below the reaction flask, the entire setup was tented in aluminum foil). The solution was decanted, concentrated in the vacuum and immediately stored in an argon atmosphere (glovebox) to mitigate reformation of the starting material. The resulting yellow oil (520 mg) was analysed by ^{1}H NMR spectroscopy in CD_3CN to be a mixture of enamine **4** (74.9 mg, 0.316 mmol, yield: 50%, 14.4 w/w%), 2,6-lutidine (372 mg, 71.6 w/w%), secondary amine **3** (39.5 mg, 7.6 w/w%), and residual acetonitrile (33.8 mg, 6.5 w/w%).

^{1}H NMR (400 MHz, CD_3CN): δ 5.93 (dq, *J* = 14.5, 1.4 Hz, 1 H, 8-H), 4.31 (dq, *J* = 14.5, 6.3 Hz, 1 H, 9-H), 3.88 (s, 2 H, 4-H), 1.63 (dd, *J* = 6.3, 1.4 Hz, 3 H, 10-H), 1.43 (s, 6 H, 7-H), 1.37 (s, 6 H, 6-H). **^{13}C{^{1}H} NMR** (101 MHz, CD_3CN): δ 171.3 (C, C-2), 127.9 (CH, C-8), 117.9 (C, C-5), 93.3 (CH, C-9), 90.8 (C, C-1), 71.1 (C, C-3), 27.7 (CH_3, C-7), 25.7 (CH_3, C-6), 16.7 (CH_3, C-10), 16.2 (CH_2, C-4).

Additional resonances in the NMR spectra were assigned to 2,6-lutidine and the secondary amine **3**:

2,6-Lutidine: ^{1}H NMR (400 MHz, CD_3CN): δ 7.49 (t, *J* = 7.7 Hz, 1 H), 6.98 (d, *J* = 7.7 Hz, 2 H), 2.43 (s, 6 H). ^{13}C{^{1}H} NMR (101 MHz, CD_3CN): δ 158.5, 137.4, 120.8, 24.5.

Secondary amine **3**: ^{1}H NMR (400 MHz, CD_3CN): δ 3.86 (s, 2 H), 1.34 (s, 6 H), 1.28 (s, 6 H). ^{13}C{^{1}H} NMR (101 MHz, CD_3CN): δ 172.3, 118.1, 90.1, 71.0, 30.9, 29.0, 17.3.

(*E*)-2-((2,2,5,5-Tetramethyl-1-styryl-2,5-dihydro-1*H*-imidazol-4-yl)thio)acetonitrile (**5**) (MJH-I-77)

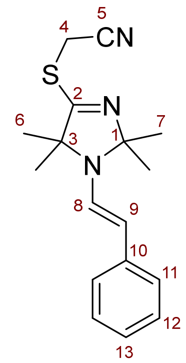

5

In a two-neck round bottom flask fitted with a Dean Stark trap a solution of secondary amine **3** (407 mg, 2.06 mmol), 2-phenylacetaldehyde (265 mg, 2.20 mmol), and *p*-toluenesulfonic acid (1 mol%) in dry toluene (5.3 mL) was heated to reflux for 2 h. Then, the reaction mixture was cooled at ambient temperature under an N_2 atmosphere. The solvent was evaporated under vacuum. The crude product was purified by flash chromatography[S4] (neutral alumina oxide, pentane/EtOAc 9:1): **5** (242 mg, yield: 39%); yellow solid, m.p. 122.5 °C; R_f = 0.27 (silica gel, pentane/EtOAc 8:1).

^{1}H NMR (400 MHz, CD_3CN): δ 7.25-7.15 (m, 4 H, 11-H and 12-H), 6.98-6.93 (m, 1 H, 13-H), 6.83 (d, *J* = 14.9 Hz, 1 H, 8-H), 5.44 (d, *J* = 14.9 Hz, 1 H, 9-H), 3.91 (s, 2 H, 4-H), 1.56 (s, 6 H, 7-H), 1.51 (s, 6 H, 6-H). **^{13}C{^{1}H} NMR** (101 MHz, CD_3CN): δ 171.2 (C, C-2), 141.1 (C, C-10), 129.3 (CH, C-11), 128.3 (CH, C-8), 124.2 (CH, C-12), 124.1 (CH, C-13), 117.8 (C, C-5), 99.6 (CH, C-9), 91.6 (C, C-1), 71.7 (C, C-3), 27.8 (CH_3, C-7), 25.9 (CH_3, C-6), 16.3 (CH_2, C-4). **IR** (ATR, neat): 2980, 2932, 2248, 1633, 1610, 1598, 1446, 1378, 1342, 1217, 1197, 1028, 941, 798, 758, 736, 695 cm^{-1}. **HRMS** (EI): *m/z* calcd for $C_{17}H_{21}N_3S^{\bullet+}$ [$M^{\bullet+}$]: 299.1451; found: 299.1445.

The crystal used for X-ray crystallographic data was prepared by the diffusion method where a sample of **5** was diluted in a vial with CH_2Cl_2 and placed in a chamber of pentane filled with N_2 gas. The sample was left to sit in the fridge (7 °C) undisturbed for 4 days. The crystal was isolated as a yellow needle and characterised by X-ray crystallography (Section 4).

4. Crystallographic data for enamine 5

The X-ray intensity data of **5** (av098) were measured on a Bruker D8 Venture TXS system equipped with a multilayer mirror monochromator and a Mo Kα rotating anode X-ray tube (λ = 0.71073 Å). The frames were integrated with the Bruker SAINT software package.[S5] Data were corrected for absorption effects using the Multi-Scan method (SADABS).[S6] The structure was solved and refined using the Bruker SHELXTL Software Package.[S7] All hydrogen atoms were calculated in ideal geometry riding on their parent atoms. The figure was drawn at the 25% ellipsoid probability level.[S8]

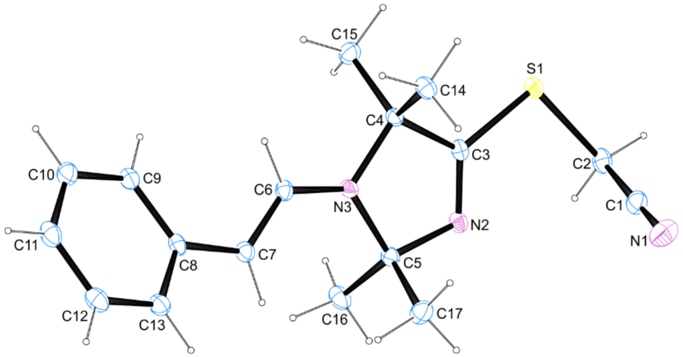

(av098)

CCDC 2254388

Crystallographic data for **5**

	**5**
net formula	$C_{17}H_{21}N_3S$
M_r/g mol^{-1}	299.43
crystal size/mm	0.150 × 0.130 × 0.040
T/K	173.(2)
radiation	MoKα
diffractometer	'Bruker D8 Venture TXS'
crystal system	monoclinic
space group	'P 1 21/c 1'
a/Å	18.5673(6)
b/Å	5.8814(2)
c/Å	15.1443(6)
α/°	90
β/°	99.7200(10)
γ/°	90
V/Å^3	1630.04(10)
Z	4
calc. density/g cm^{-3}	1.220
μ/mm^{-1}	0.196
absorption correction	Multi-Scan
transmission factor range	0.95–0.99
refls. measured	27725
R_{int}	0.0415
mean σ(*I*)/*I*	0.0257
θ range	3.117–27.482
observed refls.	3312
x, *y* (weighting scheme)	0.0347, 0.7093
hydrogen refinement	constr
Flack parameter	-
refls in refinement	3740
parameters	194
restraints	0
$R(F_{obs})$	0.0359
$R_w(F^2)$	0.0924
S	1.104
shift/error$_{max}$	0.001
max electron density/e Å^{-3}	0.276
min electron density/e Å^{-3}	−0.217

5. Reactions of enamines 4 or 5 with benzhydrylium tetrafluoroborates 7

3,3-Bis(4-(dimethylamino)phenyl)-2-methylpropanal 9a (MJH-I-119)

In a separate flask, 220 mg of a mixture containing enamine **4**, 2,6-lutidine, secondary amine **3**, and acetonitrile was weighed, in which 47.4 mg of this mixture was estimated to be **4** based on the relative integral heights the ^{1}H NMR spectrum of the sample (MJH-I-116, not shown).

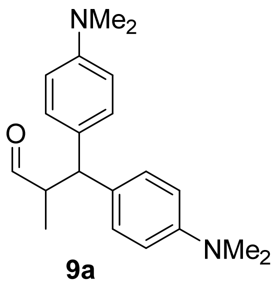

9a

To a solution of **7d** (52.2 mg, 0.153 mmol) in acetonitrile (3.4 mL) was added **4** (47.4 mg, 0.200 mmol) in acetonitrile (3.6 mL) dropwise at room temperature. The reaction was stirred at room temperature for 2 h. Most of the solvent was then evaporated under vacuum. The resulting oil was hydrolised by adding 2 M aq HCl (3 mL) and stirring of the solution at room temperature for 30 min. The solution was neutralised using 2 M aq NaOH. Subsequently, the solution was mixed with CH_2Cl_2 (10 mL), and the aqueous layer was extracted with CH_2Cl_2 (3 × 20 mL). The combined organic phases were dried over $MgSO_4$, and the volatiles were evaporated under vacuum. The crude product was purified by flash chromatography (silica gel, pentane/EtOAc 6:1) to give **9a** (30.1 mg, yield: 63%) as a light pink, flaky solid; R_f = 0.69 (silica gel, CH_2Cl_2/Et_2O 3:1). ^{1}H and ^{13}C NMR data agree with those reported previously.[S9]

^{1}H NMR (400 MHz, $CDCl_3$) δ 9.57 (d, *J* = 3.4 Hz, 1 H), 7.15-7.08 (m, 4 H), 6.66 (t, *J* = 8.6 Hz, 4 H), 3.90 (d, *J* = 10.9 Hz, 1 H), 3.18 (dqd, *J* = 10.4, 6.8, 3.4 Hz, 1 H), 2.90 (s, 6 H), 2.88 (s, 6 H), 1.03 (d, *J* = 6.8 Hz, 3 H). **^{13}C{^{1}H} NMR** (101 MHz, $CDCl_3$) δ 205.4, 149.3, 131.1, 128.8, 128.7, 113.1, 113.0, 51.8, 50.7, 40.82, 40.78, 13.9.

2-Methyl-3,3-bis(4-(pyrrolidin-1-yl)phenyl)propanal 9b (MJH-II-3)

In a separate flask, 393 mg of a mixture containing **4**, 2,6-lutidine, secondary amine **3**, and acetonitrile was weighed, in which 56.3 mg of this mixture was estimated to be **4** based on the relative integral heights the ^{1}H NMR spectrum of the sample (MJH-I-183, shown in Figure S3).

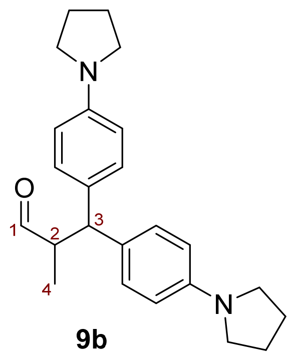

9b

To a solution of **7e** (82.2 mg, 0.209 mmol) in acetonitrile (4.1 mL) was added **4** (56.3 mg, 0.237 mmol) in acetonitrile (4.1 mL) in one portion at room temperature, at which point the reaction mixture changed colour from blue to brown. After 40 min, to the solution was added 2 M aq HCl (3 mL). This mixture was left to stir at room temperature for 30 min. Subsequently, the solution was mixed with CH_2Cl_2 (10 mL) and then washed with aq saturated $NaHCO_3$ solution. The organic layer was separated, and the aqueous layer was extracted with CH_2Cl_2 (3 x 20 mL). The combined organic layers were dried over $MgSO_4$, and the volatiles were evaporated under vacuum. The crude product was purified by flash chromatography (silica gel, loaded with CH_2Cl_2, eluent: pentane/EtOAc 9:1) to give **9b** (52.8 mg, yield: 70%) as a lavender solid; m.p. 141.7 °C; R_f = 0.44 (silica gel, pentane/Et_2O 4:1).

^{1}H NMR (800 MHz, $CDCl_3$): δ 9.58 (d, *J* = 3.4 Hz, 1 H, 1-H), 7.12 (d, *J* = 8.6 Hz, 2 H), 7.09 (d, *J* = 8.6 Hz, 2 H), 6.50 (d, *J* = 8.6 Hz, 2 H), 6.48 (d, *J* = 8.7 Hz, 2 H), 3.90 (d, *J* = 10.8 Hz, 1 H, 3-H), 3.25-3.22 (m, 8 H), 3.19-3.15 (m, 1 H, 2-H), 1.98-1.95 (m, 8 H), 1.04 (d, *J* = 6.8 Hz, 3 H, 4-H). **^{13}C{^{1}H} NMR** (201 MHz, $CDCl_3$) δ 205.7 (CH, C-1), 146.61 (C), 146.58 (C), 129.92 (C), 129.86 (C), 128.86 (CH), 128.79 (CH), 111.9 (CH), 111.8 (CH), 51.9 (CH, C-3), 50.7 (CH, C-2), 47.70 (CH_2), 47.67 (CH_2), 25.57 (CH_2), 25.56 (CH_2), 13.9 (CH_3, C-4). **IR** (ATR, neat) 2965, 2927, 2821, 1717, 1612, 1516, 1486, 1460, 1365, 1184, 1060, 965, 800 cm^{-1}. **HRMS** (EI): *m/z* calcd for $C_{24}H_{30}N_2O^{\bullet+}$ [$M^{\bullet+}$]: 362.2353; found: 362.2354.

2-Methyl-3,3-bis(1-methylindolin-5-yl)propanal 9c (MJH-II-23)

In a separate flask, 647 mg of a mixture containing **4**, 2,6-lutidine, secondary amine **3**, and acetonitrile was weighed, in which 99.7 mg of this mixture was estimated to be **4** based on the relative integral heights the ^{1}H NMR spectrum of the sample (MJH-I-124, shown in Figure S4).

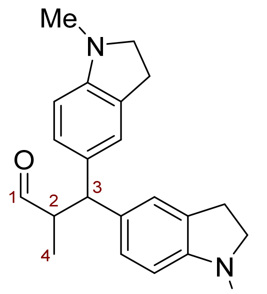

9c

To a solution of **7g** (80.3 mg, 0.220 mmol) in acetonitrile (2.4 mL) was added **4** (99.7 mg, 0.420 mmol) in acetonitrile (2.2 mL) in one portion at room temperature, at which point the reaction mixture changed colour from blue to green. After 1 h, to the solution was added 2 M aq HCl (3 mL). This mixture was left to stir at room temperature for 30 min. Subsequently, the solution was mixed with CH_2Cl_2 (10 mL) and then washed with aq saturated $NaHCO_3$ solution. The organic layer was separated, and the aqueous layer was extracted with CH_2Cl_2 (3 x 20 mL). The combined organic layers were dried over $MgSO_4$, and the volatiles were evaporated under vacuum. The crude product was purified by flash chromatography (silica gel, CH_2Cl_2/Et_2O 6:1) to furnish **9c** (54.6 mg, yield: 74%) as a colorless oil; R_f = 0.50 (silica gel, CH_2Cl_2/Et_2O 4:1).

^{1}H NMR (600 MHz, $CDCl_3$): δ 9.53 (d, *J* = 3.5 Hz, 1 H, 1-H), 6.98-6.93 (m, 4 H), 6.39 (d, *J* = 7.9 Hz, 1 H), 6.37 (d, *J* = 7.9 Hz, 1 H), 3.84 (d, *J* = 11.1 Hz, 1 H, 3-H), 3.26-3.22 (m, 4 H), 3.13 (dqd, *J* = 10.4, 6.8, 3.5 Hz, 1 H, 2-H), 2.90-2.85 (m, 4 H), 2.71 (s, 3 H), 2.70 (s, 3 H), 1.00 (d, *J* = 6.8 Hz, 3 H, 4-H). **^{13}C{^{1}H} NMR** (201 MHz, $CDCl_3$) δ 205.6 (CH, C-1), 152.2 (C), 152.1 (C), 132.57 (C), 132.56 (C), 131.04 (C), 130.96 (C), 127.0 (CH), 126.8 (CH), 124.2 (CH), 124.0 (CH), 107.2 (CH), 107.1 (CH), 56.4 (CH_2), 56.3 (CH_2), 52.7 (CH, C-3), 50.9 (CH, C-2), 36.5 (CH_3), 36.4 (CH_3), 28.91 (CH_2), 28.87 (CH_2), 14.0 (CH_3, C-4); **IR** (ATR, neat) 2950, 2924, 2851, 2806, 1720, 1613, 1495, 1470, 1452, 1376, 1267, 1213, 1182, 1082, 989, 890, 808, 732, 701 cm^{-1}. **HRMS** (EI): *m/z* calcd for $C_{22}H_{26}N_2O^{\bullet+}$ [$M^{\bullet+}$]: 334.2040; found: 334.2041.

3,3-Bis(4-morpholinophenyl)-2-phenylpropanal 9d (MJH-II-15)

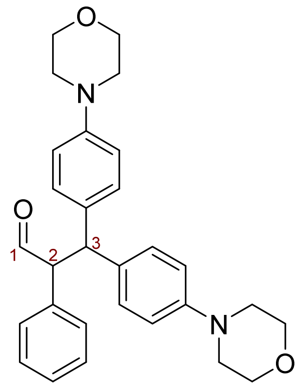

9d

To a solution of **7c** (99.1 mg, 0.234 mmol) in acetonitrile (4.2 mL) was added enamine **5** (63.1 mg, 0.211 mmol) in acetonitrile (4.2 mL) in one portion at room temperature. The solution turned dark purple as the blue solution stirred at room temperature. After 20 min, 2 M aq HCl (3 mL) was added. This mixture was left to stir at room temperature for 30 min. Subsequently, the solution was mixed with CH_2Cl_2 (10 mL) and then washed with aq saturated $NaHCO_3$ solution. The organic layer was separated, and the aqueous layer was extracted with CH_2Cl_2 (3 x 20 mL). The combined organic layers were dried over $MgSO_4$, and the volatiles were evaporated under vacuum. The crude product (dark purple oil) was purified by flash chromatography (silica gel, CH_2Cl_2/Et_2O 6:1), and the dark purple product was isolated in a vial and diluted in CH_2Cl_2. The vial was placed in a diffusion chamber with pentane. After 3 days, the mother liquor was decanted, and the solvent evaporated under vacuum. Aldehyde **9d** (69.2 mg, yield: 72%) was isolated as a colourless powder; m.p. 87.4 °C; R_f = 0.34 (3:1, CH_2Cl_2/Et_2O, SiO_2).

^{1}H NMR (400 MHz, $CDCl_3$): δ 9.64 (d, *J* = 3.7 Hz, 1 H, 1-H), 7.28-7.14 (m, 7 H), 6.98 (d, *J* = 8.7 Hz, 2 H), 6.84 (d, *J* = 8.7 Hz, 2 H), 6.64 (d, *J* = 8.7 Hz, 2 H), 4.65 (d, *J* = 12.0 Hz, 1 H, 3-H), 4.39 (dd, *J* = 11.9, 3.7 Hz, 1 H, 2-H), 3.84-3.82 (m, 4 H), 3.78-3.76 (m, 4 H), 3.12-3.09 (m, 4 H), 3.02-2.99 (m, 4 H). **^{13}C{^{1}H} NMR** (101 MHz, $CDCl_3$): δ 199.5 (CH, C-1), 150.0 (C), 149.4 (C), 135.0 (C), 133.9 (C), 133.5 (C), 129.5 (CH), 129.00 (CH), 128.95 (CH, CH), 127.6 (CH), 116.1 (CH), 115.6 (CH), 67.1 (CH_2), 67.0 (CH_2), 63.7 (CH, C-2), 50.4 (CH, C-3), 49.33 (CH_2), 49.30 (CH_2). **IR** (ATR, neat) 3030, 2959, 2852, 2819, 1720, 1681, 1610, 1512, 1448, 1379, 1332, 1302, 1228, 1118, 1051, 925, 814, 755, 699 cm^{-1}. **HRMS** (EI): *m/z* calcd for $C_{29}H_{32}N_2O_3^{\bullet+}$ [$M^{\bullet+}$]: 456.2407. Found: 456.2403.

6. Evidence corroborating the formation of enamine **6** and NMR reaction monitoring

6.1 Formation of enamine **6** under photoirradiation

4-(4-((Cyanomethyl)thio)-2,2,5,5-tetramethyl-2,5-dihydro-1H-imidazol-1-yl)-3-methylbut-3-enenitrile 6 (MJH-III-3)

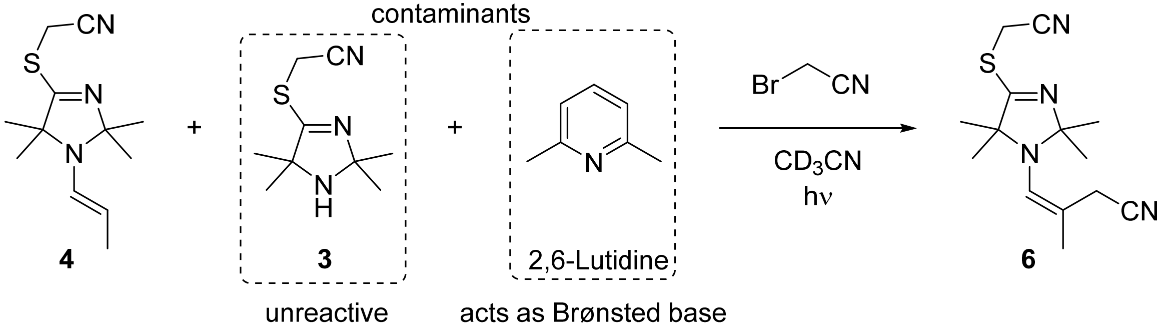

In an inert argon atmosphere (glovebox), a 31.5 mg sample of a mixture containing enamine **4**, 2,6-lutidine, secondary amine **3**, and acetonitrile was weighed in an NMR tube, in which 10.8 mg (0.046 mmol) of this mixture was **4** based on the relative integral heights the ^{1}H NMR spectrum of the sample (the ^{1}H NMR spectrum of this mixture is shown on page S20, MJH-II-181).

To the mixture in the NMR tube was added bromoacetonitrile (98.1 mg, 0.818 mmol) and CD_3CN. The NMR cap was added, and the tube was removed from the glovebox. The pale-yellow solution in the NMR tube was irradiated with a Roithner LaserTechnik-H2A1-H365 emitter (365 nm) for 1 h. During this time, the solution turned to a bright yellow colour. NMR spectroscopy indicated that enamine **4** was consumed completely and enamine **6** was formed. This solution was used without further purification to characterise **6** by NMR spectroscopy.

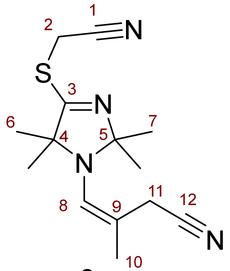

6

^{1}H NMR (400 MHz, CD_3CN) δ 5.87 (h, *J* = 1.5 Hz, 1 H, 8-H), 3.88 (s, 2 H, 2-H), 3.19 (d, *J* = 0.7 Hz, 2 H, 11-H), 1.83 (d, *J* = 1.5 Hz, 3 H, 10-H), 1.27 (s, 6 H, 7-H), 1.19 (s, 6 H, 6-H); **^{13}C {^{1}H} NMR** (101 MHz, CD_3CN) δ 171.5 (C, C-3), 132.8 (C, C-9), 127.6 (CH, C-8), 119.0 (C, C-12), 117.8 (C, C-1), 92.1 (C, C-5), 73.0 (C, C-4), 27.7 (CH_3, C-7), 25.6 (CH_3, C-6), 24.6 (CH_2, C-11), 16.2 (CH_2, C-2), 16.1 (CH_3, C-10).

Additional resonances in the NMR spectra were assigned to 2,6-lutidine, the secondary amine **3**, and bromoacetonitrile:

2,6-Lutidine: ^{1}H NMR (400 MHz, CD_3CN): δ 7.71 (t, *J* = 7.8 Hz, 1 H), 7.15 (d, *J* = 7.7 Hz, 2 H), 2.56 (s, 6 H); ^{13}C{^{1}H} NMR (101 MHz, CD_3CN) 157.1, 140.1, 122.4, 22.8.

Secondary amine **3**: ^{1}H NMR (400 MHz, CD_3CN): δ 3.87 (s, 2 H), 1.38 (s, 6 H), 1.32 (s, 6 H); ^{13}C{^{1}H} NMR (101 MHz, CD_3CN) δ 172.1, 117.9, 90.3, 71.1, 30.7, 28.7, 17.3.

Bromoacetonitrile (BAN): ^{1}H NMR (400 MHz, CD_3CN): δ 3.98 (s, 2 H); ^{13}C{^{1}H} NMR (101 MHz, CD_3CN) δ 116.7, 7.6.

4-(4-((Cyanomethyl)thio)-2,2,5,5-tetramethyl-2,5-dihydro-1H-imidazol-1-yl)-3-methylbut-3-enenitrile (6)

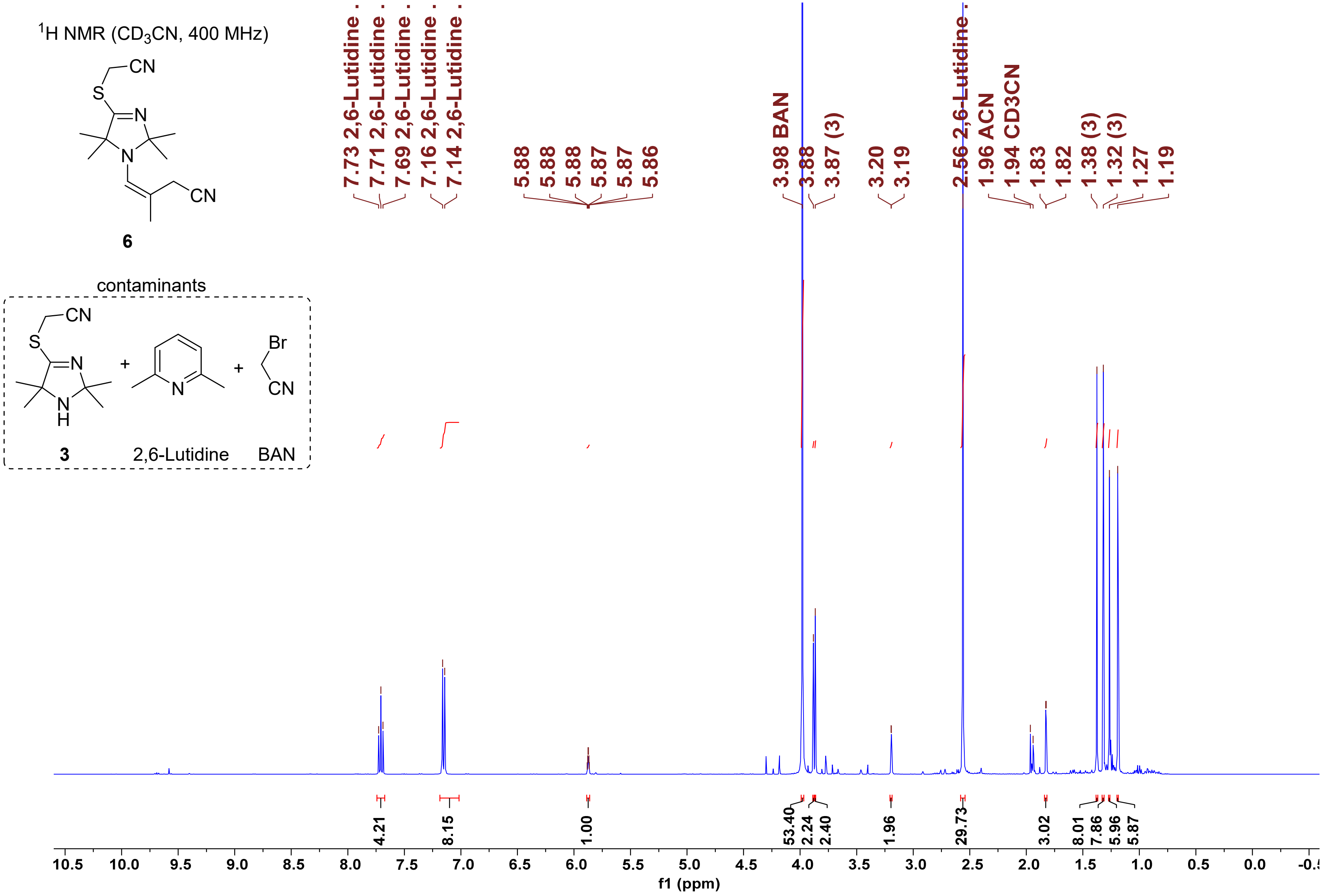

4-(4-((Cyanomethyl)thio)-2,2,5,5-tetramethyl-2,5-dihydro-1H-imidazol-1-yl)-3-methylbut-3-enenitrile (6)

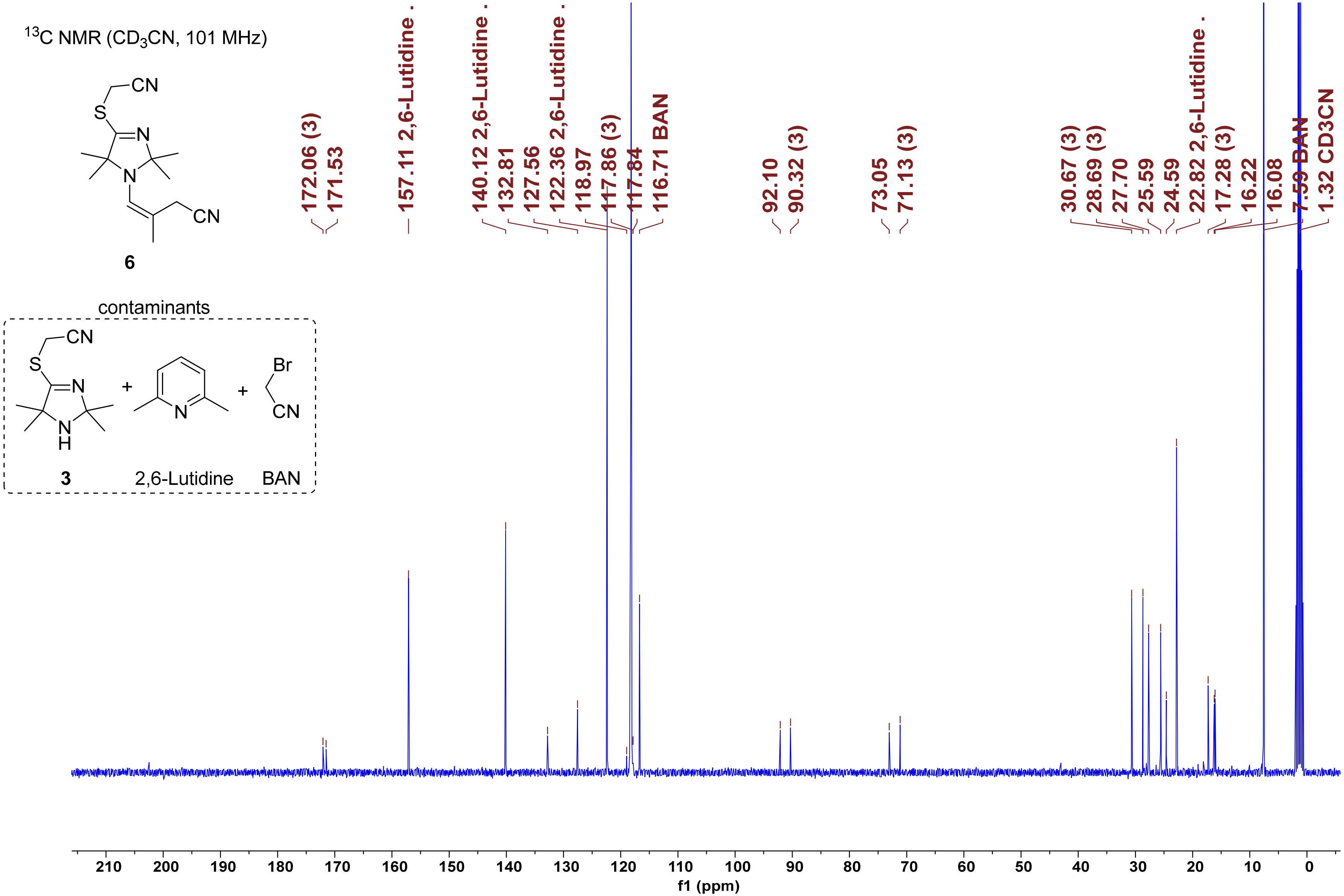

Initial mixture of enamine **4** with (unreactive) secondary amine **3** and 2,6-lutidine

^{1}H NMR (CD_3CN, 400 MHz) (MJH-II-181)

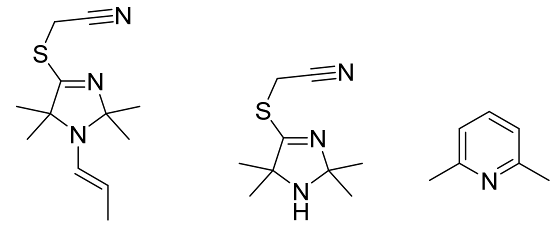

4 **3** 2,6-Lutidine

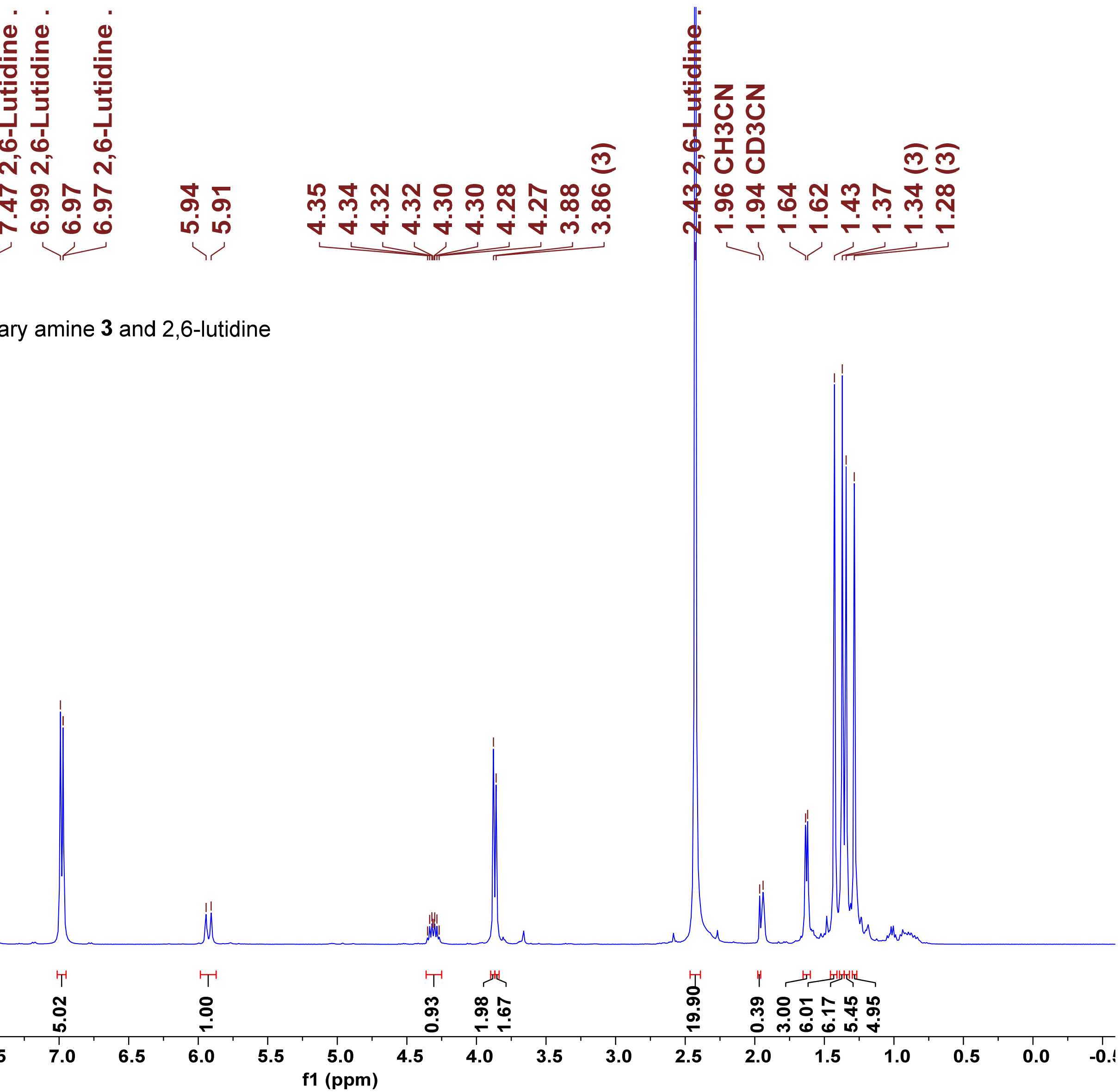

6.2 Formation of enamine **6** in the dark

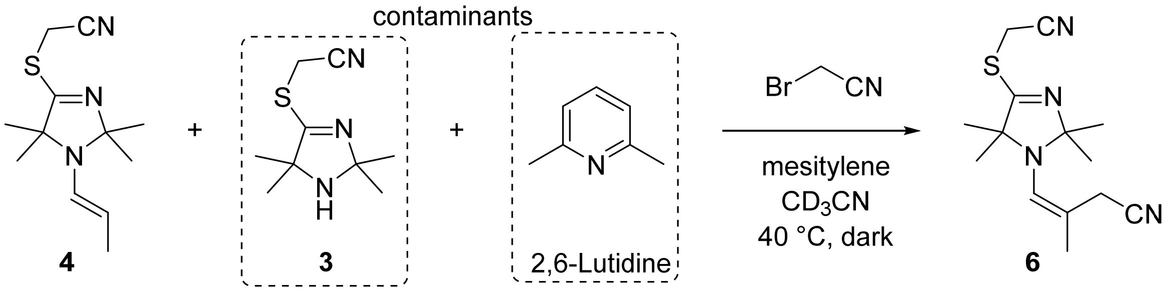

(MJH-III-6)

A 63.2 mg mixture of enamine **4**, 2,6-lutidine, secondary amine **3**, and acetonitrile was weighed in an NMR tube in an argon atmosphere (glovebox), in which 21.5 mg (0.091 mmol) of this mixture was **4** based on the relative integral heights the ^{1}H NMR spectrum of the sample (the ^{1}H NMR spectrum of this mixture is shown on page S20, MJH-II-181).

To the mixture in the NMR tube was added bromoacetonitrile (102 mg, 0.850 mmol), mesitylene (10.6 mg, 0.0882 mmol), and CD_3CN. The NMR cap was added, and the tube was removed from the glovebox. The NMR tube was wrapped in aluminium foil and heated in an oil bath at 40 °C. The foil was only removed from the NMR tube when traveling to and from the NMR spectrometer. The reaction was monitored by NMR spectroscopy over the course of 23 h.

Table S2. Decrease in enamine **4** concentration and concurrent increase in the α-branched enamine **6** concentration over time (CD_3CN, 40 °C, dark, full spectra are shown in Figure S2) as exemplified by the change of the resonances for 10-H and 11-H of **6** and 10-H of **4**. Concentrations of enamines **4** and **6** were calculated based on the integration of the mesitylene internal standard at δ 6.77 ppm (10.6 mg, 0.0882 mmol).

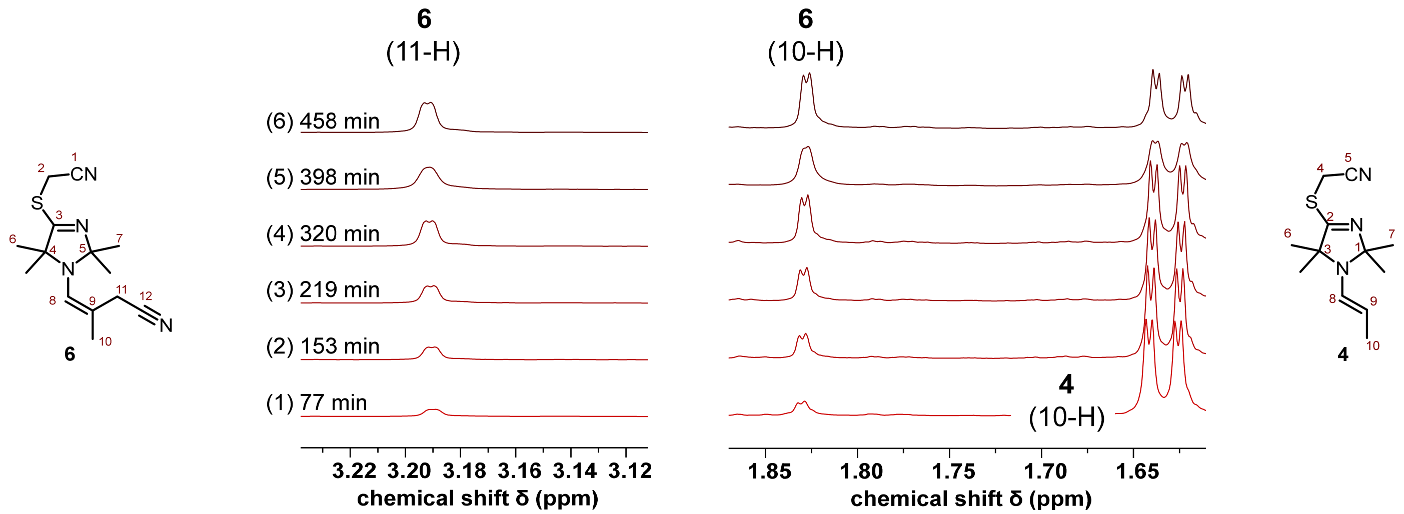

Spectra	*t* (min)	Integration mesitylene (*δ 6.77, 3H)*	Integration **4** (*δ 1.63, 3 H, 10-H)*	Integration **6** (*δ 1.82, 3 H, 10-H)*	**4** (mmol)	**6** (mmol)
1	77	1.00	0.93	0.09	0.0820	0.0081
2	153	1.00	0.83	0.13	0.0732	0.0117
3	219	1.00	0.74	0.18	0.0653	0.0162
4	320	1.00	0.72	0.25	0.0635	0.0225
5	398	1.00	0.62	0.29	0.0547	0.0261
6	458	1.00	0.58	0.33	0.0512	0.0297
7	1385	1.00	0.04[a]	0.52	0.0106[a]	0.0467

[a] As minor side products were present at the location of the 10-H resonance of enamine **4** *(*δ 1.63 ppm) after 23 hours, the concentration of enamine **4** at *t* = 1385 min was calculated based on the 8-H peak for enamine **4** (δ 5.93 ppm, 1 H).

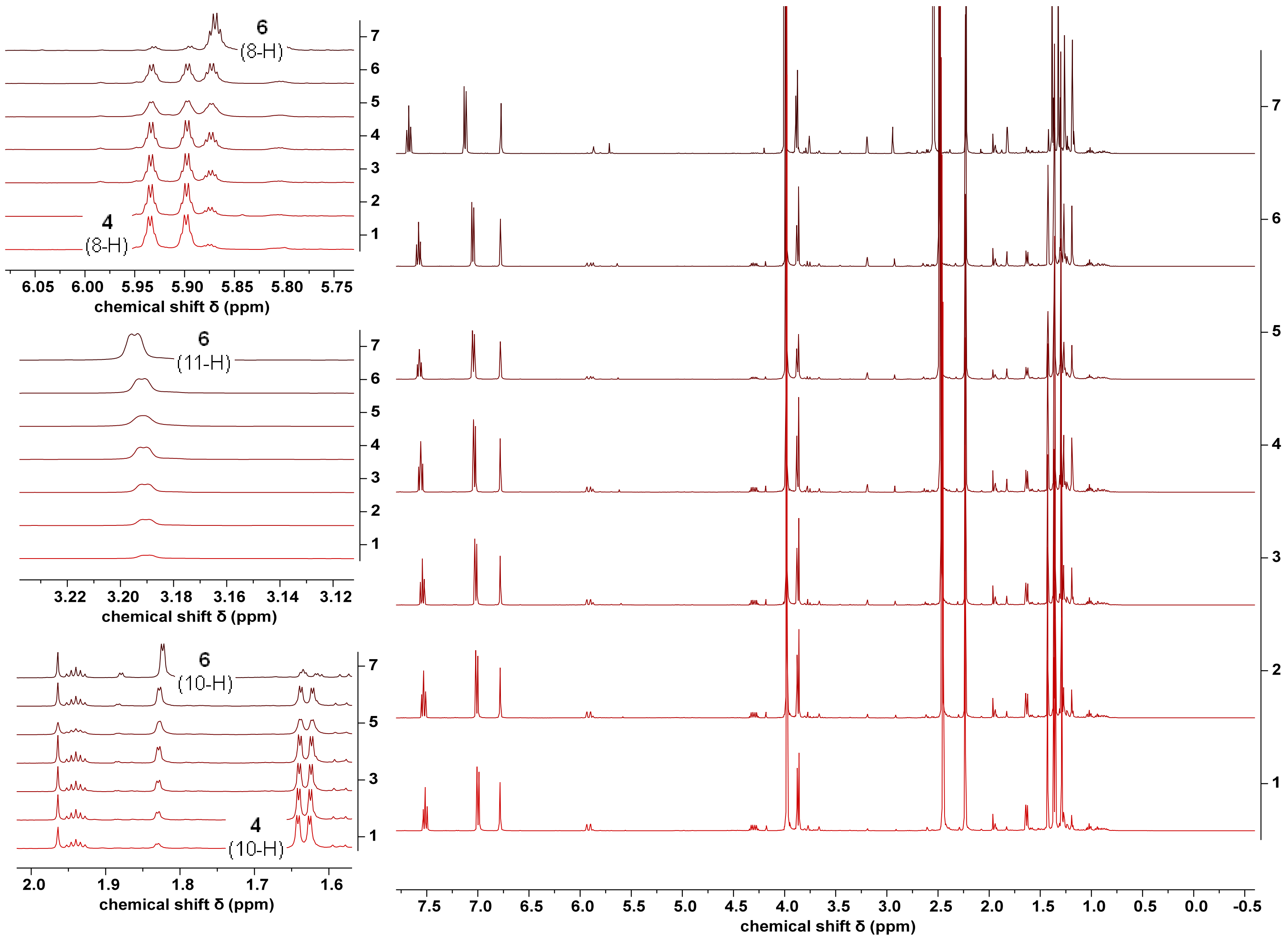

Figure S2. Online ^{1}H NMR monitoring of the reaction of enamine **4** with bromoacetonitrile in the dark to give the enamine **6** (CD_3CN, 40 °C, dark).

7. (TIM 3)-catalysed reaction (under irradiation with 365 nm light)

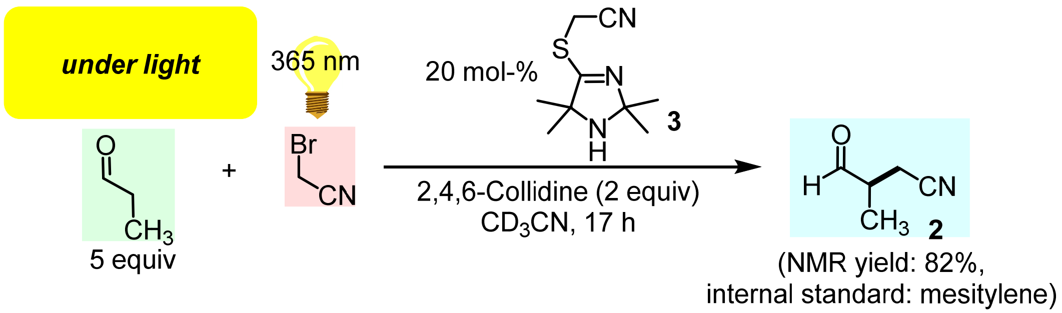

(MJH-III-153)

To an NMR tube was added propanal (80 µL, 1.12 mmol), bromoacetonitrile (26.6 mg, 0.222 mmol), 2,4,6-collidine (59 µL, 0.446 mmol), and the secondary amine TIM **3** (8.6 mg, 0.044 mmol) in CD_3CN (0.5 mL). The solution was slightly yellow and clear. The tube was then irradiated with a Roithner LaserTechnik-H2A1-H365 emitter (365 nm) placed below for 17 h. At this point large crystalline white solids accumulated in the NMR tube, but the solution remained slightly yellow. To the solution was added additional CD_3CN (approximately 0.5 mL, the white solids did not dissolve) and then an ^{1}H NMR spectrum was recorded to confirm the consumption of bromoacetonitrile (MJH-III-153.1). To the NMR tube was added mesitylene (13.8 mg, 0.115 mmol) as an internal integration standard, and the yield of **2** was determined to be 82%. NMR spectroscopic data for **2** agree with those reported previously.[S10]

Aldehyde **2:** **^{1}H NMR** (400 MHz, CD_3CN): δ 9.58 (s, 1 H), 2.84-2.76 (m, 1 H), 2.63 (dd, J = 17.0, 6.1 Hz, 1 H), 2.54 (dd, J = 17.0, 6.5 Hz, 1 H), 1.24 (d, J = 7.5 Hz, 3 H); **^{13}C{^{1}H} NMR** (101 MHz, CD_3CN) δ 202.5 (CH), 119.4 (C_q), 43.1 (CH), 18.1 (CH_2), 13.2 (CH_3).

Additional resonances in the mixture were assigned to (a) propanal: ^{1}H NMR (400 MHz, CD_3CN): δ 9.70 (t, J = 1.2 Hz, 1 H), 2.42 (dq, J = 7.4, 1.2 Hz, 2 H), 1.01 (t, J = 7.4 Hz, 3 H); ^{13}C NMR (101 MHz, CD_3CN) δ 204.2 (CH), 37.6 (CH_2), 6.2 (CH_3); (b) 2,4,6-collidine: ^{1}H NMR (400 MHz, CD_3CN): δ 6.90 (s, 2 H), 2.43 (s, 6 H), 2.26 (s, 3 H); ^{13}C NMR (101 MHz, CD_3CN) δ 157.2 (C_q), 150.8 (C_q), 122.7 (CH), 23.1 (CH_3), 21.0 (CH_3); and (c) mesitylene (internal standard) : ^{1}H NMR (400 MHz, CD_3CN): δ 6.78 (s, 3 H), 2.23 (s, 9 H); ^{13}C NMR (101 MHz, CD_3CN) δ 138.6 (C_q), 127.6 (CH), 21.2 (CH_3).

MJH-III-153.1 (^{1}H NMR, 400 MHz, CD_3CN)

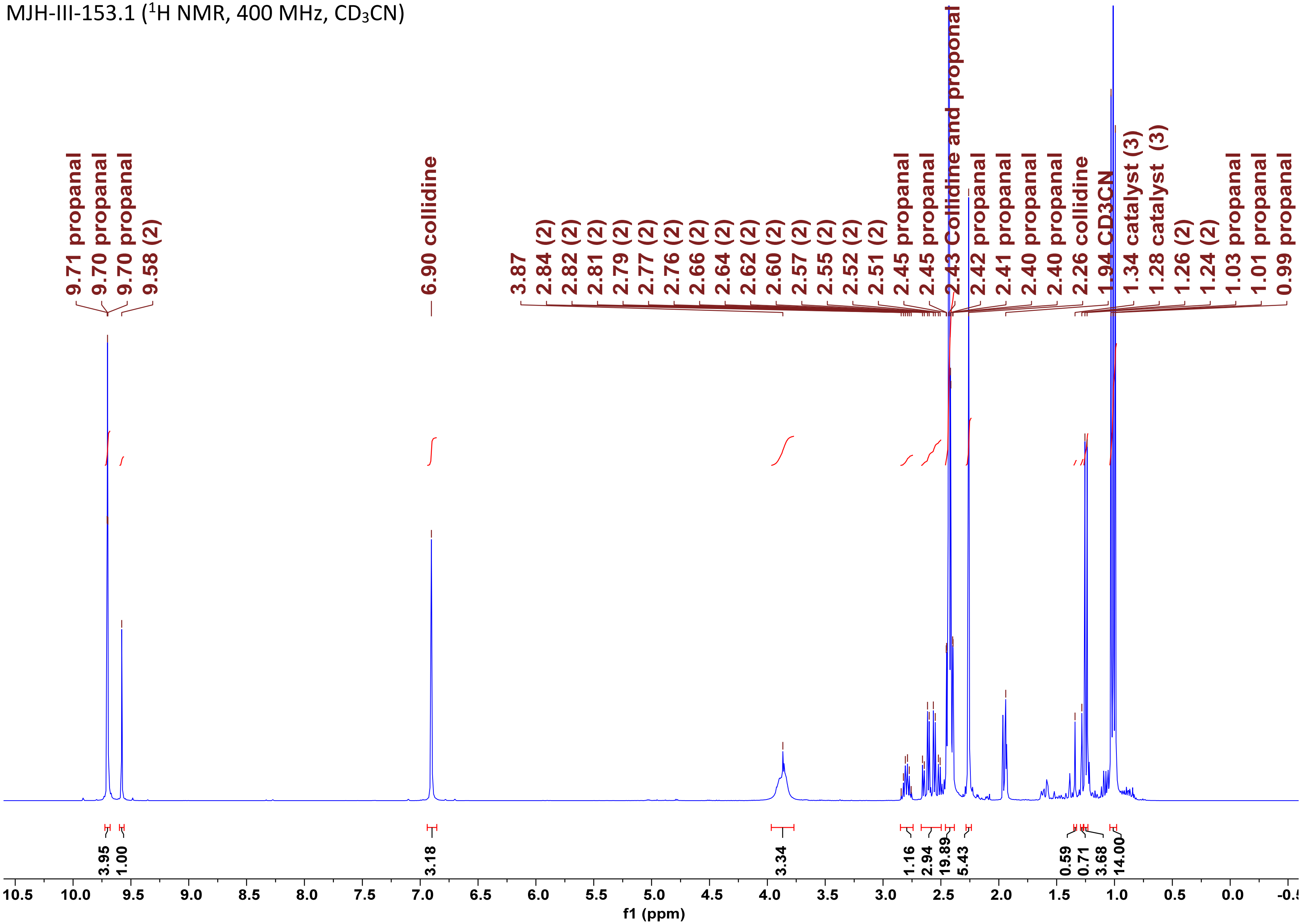

Resonances in MJH-III-153.1, which were assigned to aldehyde **2**

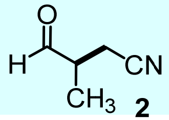

2

Sections of MJH-III-153.1 (^{1}H NMR, 400 MHz, CD_3CN)

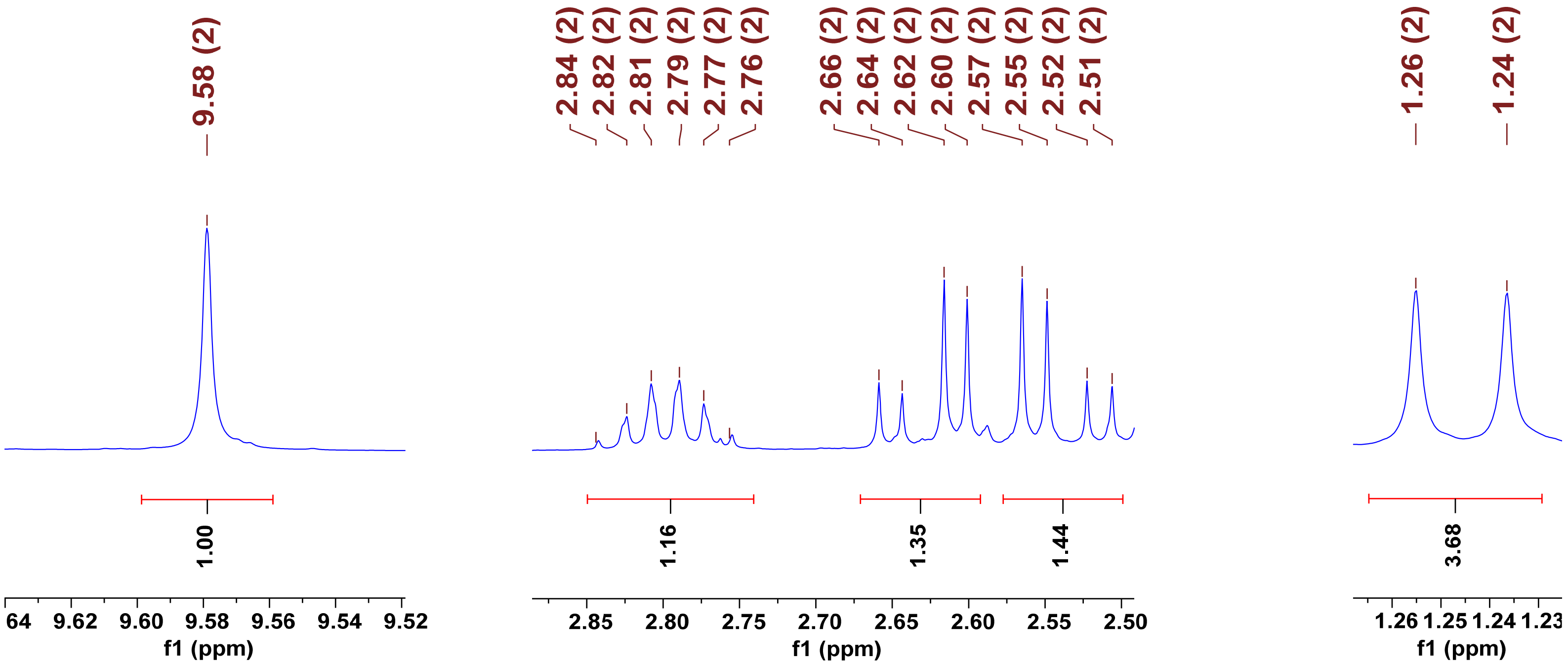

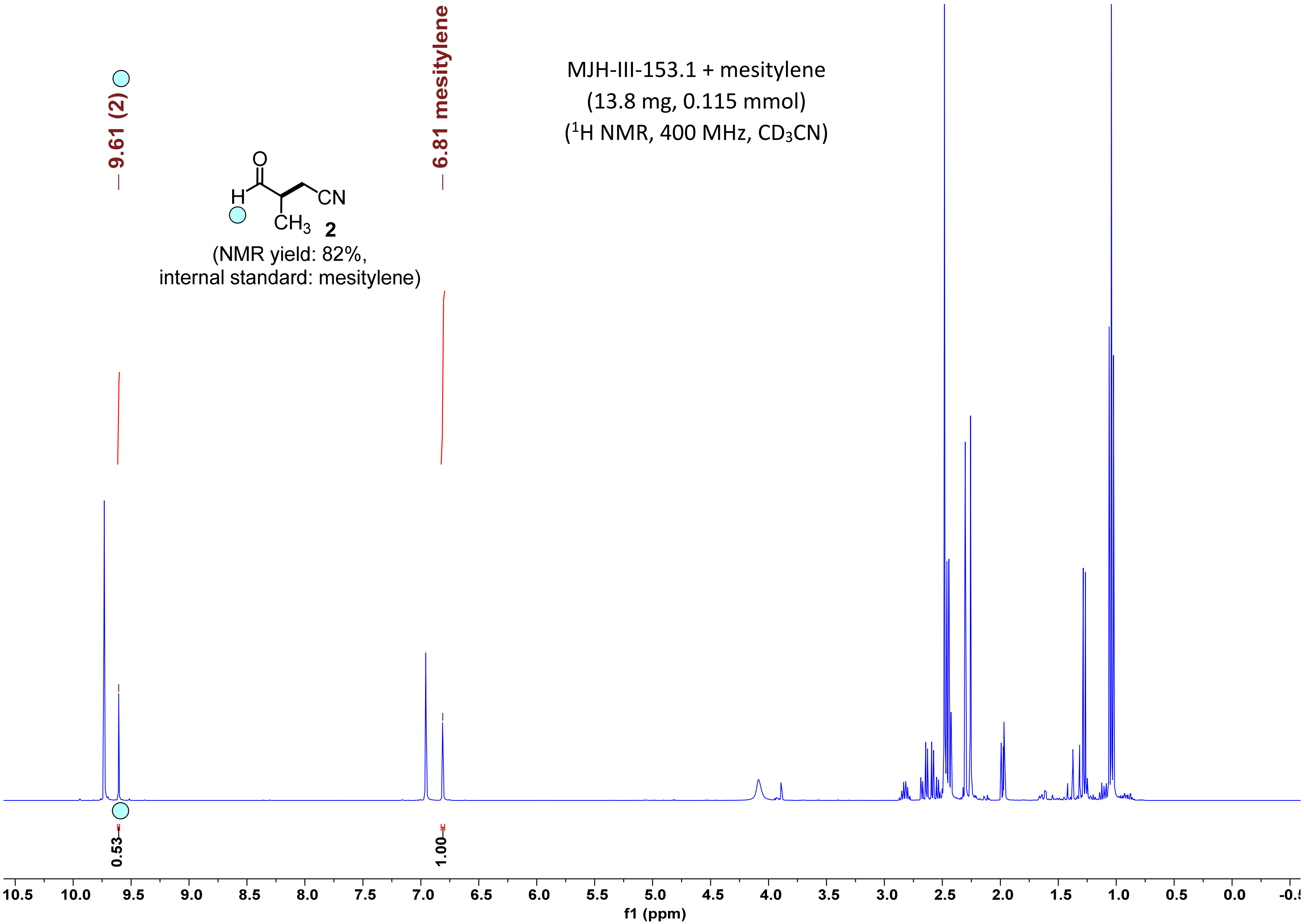

9.61 (2)
6.81 mesitylene
MJH-III-153.1 + mesitylene
(13.8 mg, 0.115 mmol)
(1H NMR, 400 MHz, CD3CN)
O
H
CN
CH3
2
(NMR yield: 82%,
internal standard: mesitylene)
0.53
1.00
10.5 10.0 9.5 9.0 8.5 8.0 7.5 7.0 6.5 6.0 5.5 5.0 4.5 4.0 3.5 3.0 2.5 2.0 1.5 1.0 0.5 0.0
f1 (ppm)

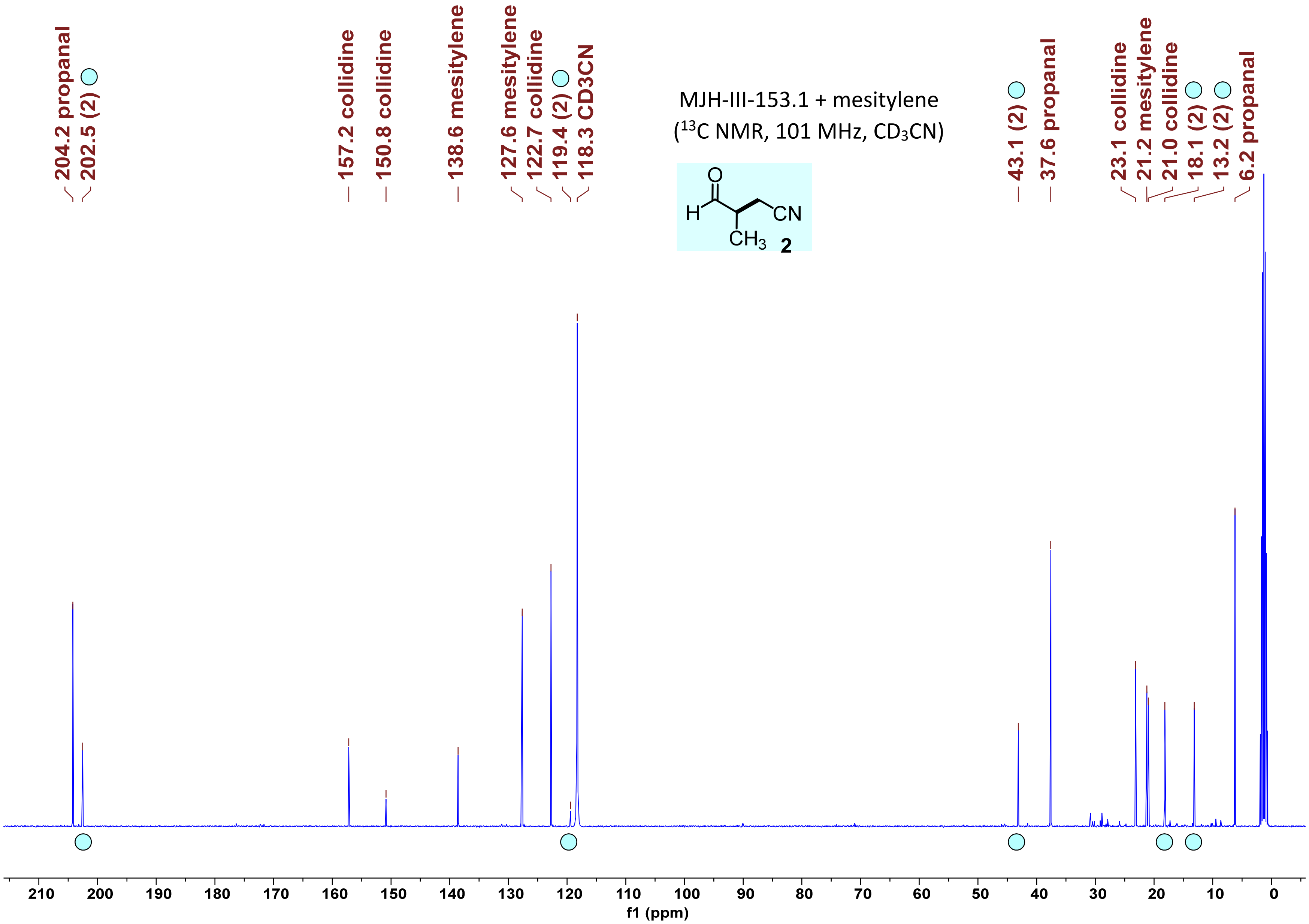
MJH-III-153.1 + mesitylene
(13C NMR, 101 MHz, CD3CN)
H
O
CN
CH3
2
204.2 propanal
202.5 (2)
157.2 collidine
150.8 collidine
138.6 mesitylene
127.6 mesitylene
122.7 collidine
119.4 (2)
118.3 CD3CN
43.1 (2)
37.6 propanal
23.1 collidine
21.2 mesitylene
21.0 collidine
18.1 (2)
13.2 (2)
6.2 propanal
210
200
190
180
170
160
150
140
130
120
110
100
90
80
70
60
50
40
30
20
10
0
f1 (ppm)

8. (TIM 3)-catalysed reaction (in the dark)

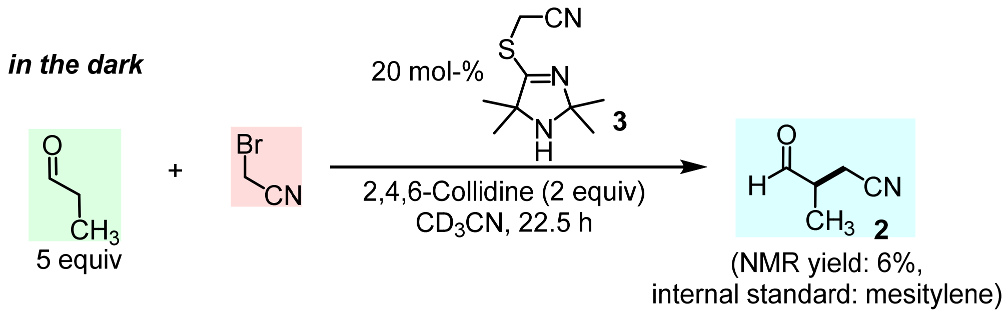

(MJH-NH343)

To an NMR tube was added propanal (80 µL, 1.12 mmol), bromoacetonitrile (26.7 mg, 0.223 mmol), 2,4,6-collidine (59 µL, 0.446 mmol), and the secondary amine TIM **3** (8.6 mg, 0.044 mmol) in CD_3CN (0.5 mL). The solution was slightly yellow and clear. The NMR tube was stored at ambient temperature in a black paper bag (to keep it in the dark). After a reaction time of 22.5 h, mesitylene (6.9 mg, 0.058 mmol) was added as an internal integration standard to the NMR sample. The yield of **2** was determined to be 6 %. NMR spectroscopic data for **2** agree with those reported in Section 9.

About 60% of the initial amount of the free catalyst TIM **3** (0.027 mmol) was still detectable in the NMR sample. The remaining 40% of the initially added cyclic amine **3** reacted with propanal to furnish enamine **4** (0.017 mmol). Only trace amounts (< 1%) of enamine **6** were detected. Further resonances in the mixture were assigned to remaining starting materials.

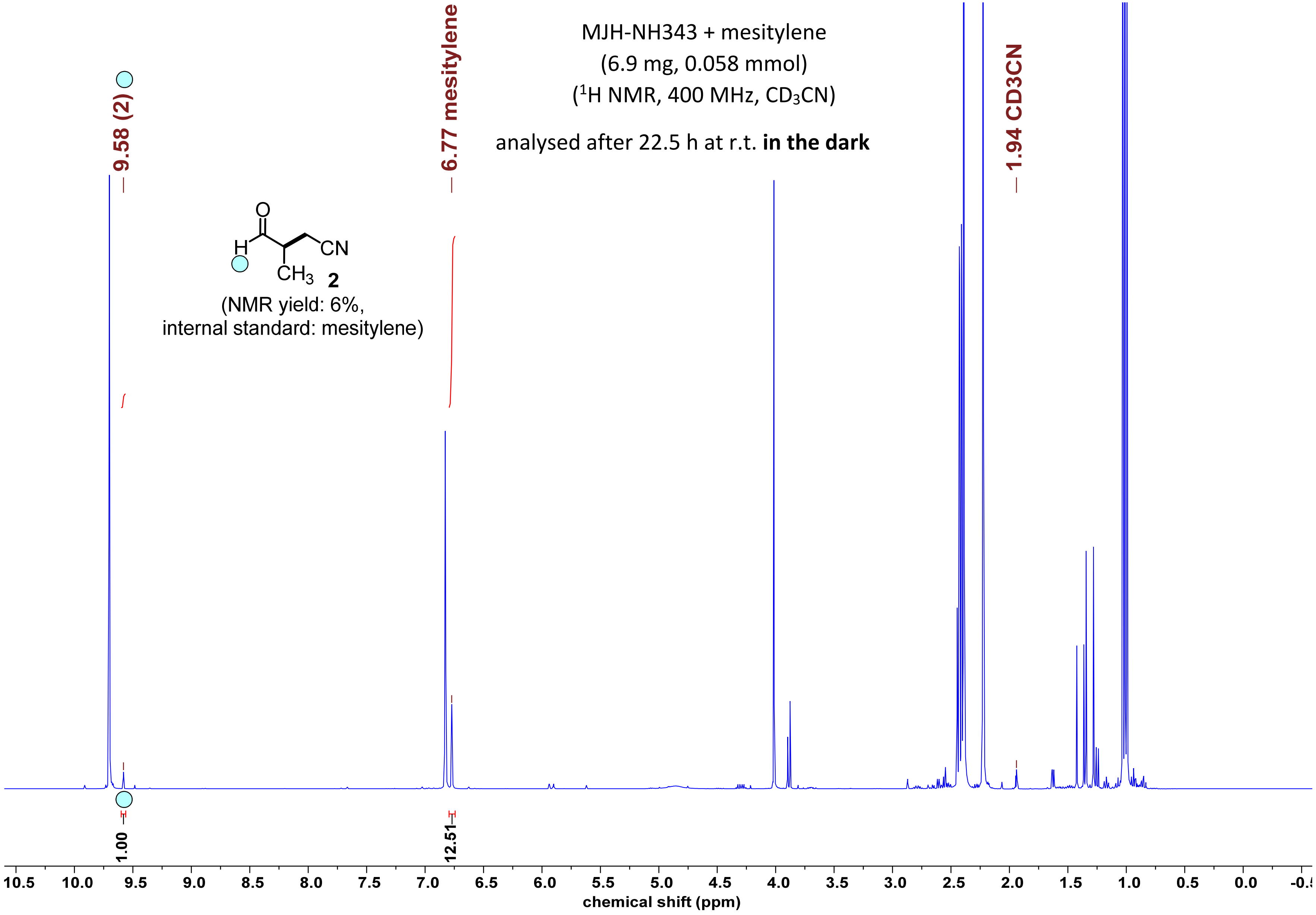

MJH-NH343 + mesitylene
(6.9 mg, 0.058 mmol)
(^{1}H NMR, 400 MHz, CD_3CN)
analysed after 22.5 h at r.t. **in the dark**
9.58 (2)
6.77 mesitylene
1.94 CD3CN
O
H
CN
CH_3
2
(NMR yield: 6%,
internal standard: mesitylene)
1.00
12.51
10.5 10.0 9.5 9.0 8.5 8.0 7.5 7.0 6.5 6.0 5.5 5.0 4.5 4.0 3.5 3.0 2.5 2.0 1.5 1.0 0.5 0.0
chemical shift (ppm)

9. Kinetic experiments

9.1 Kinetics of the propanal-derived enamine **4** in acetonitrile

Table S3. Kinetics of the reactions of **4** with **7d** in CH_3CN at 20 °C (stopped-flow, λ = 606 nm)

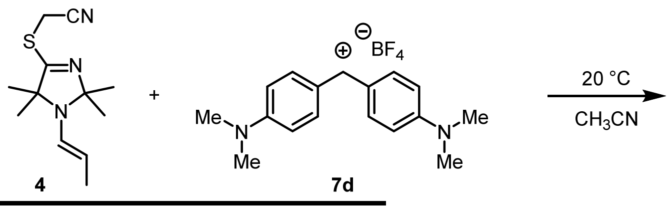

[**4**][a] (M)	[**7d**] (M)	k_{obs} (s^{-1})
2.90×10^{-4}		1.90×10^{-2}
5.74×10^{-4}	2.38×10^{-5}	3.88×10^{-2}
8.64×10^{-4}		5.97×10^{-2}
1.15×10^{-3}		7.96×10^{-2}

$k_2 = 7.06 \times 10^{1}\ M^{-1}\ s^{-1}$

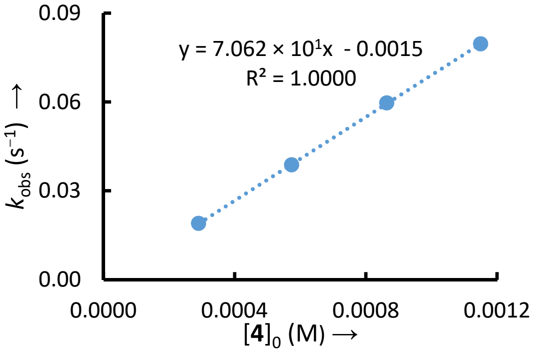

[a] The content of **4** in a mixture containing 2,6-lutidine, **3**, **4**, and acetonitrile (total: 165.4 mg) was determined to be 23.7 mg (see Figure S3 for the ^{1}H NMR spectrum of the sample). This mass was used to calculate the concentrations of the enamine **4.**

Table S4. Kinetics of the reactions of **4** with **7e** in CH_3CN at 20 °C (stopped-flow, λ = 612 nm)

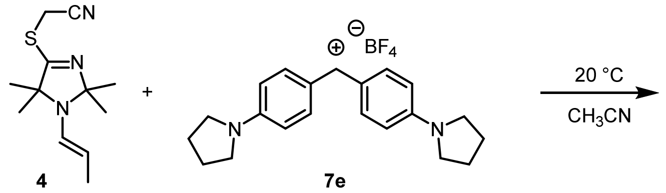

[**4**][a] (M)	[**7e**] (M)	k_{obs} (s^{-1})
2.90×10^{-4}		5.82×10^{-3}
5.74×10^{-4}	2.51×10^{-5}	1.18×10^{-2}
8.64×10^{-4}		1.80×10^{-2}
1.15×10^{-3}		2.42×10^{-2}

$k_2 = 2.14 \times 10^{1}\ M^{-1}\ s^{-1}$

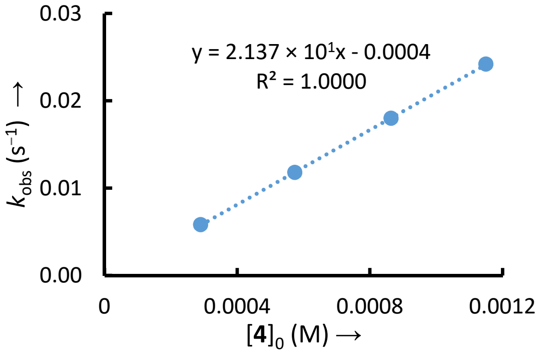

[a] The content of **4** in a mixture containing 2,6-lutidine, **3**, **4**, and acetonitrile (total: 165.4 mg) was determined to be 23.7 mg (see Figure S3 for the ^{1}H NMR spectrum of the sample). This mass was used to calculate the concentrations of the enamine **4.**

Table S5. Kinetics of the reactions of **4** with **7f** in CH_3CN at 20 °C (stopped-flow, λ = 620 nm)

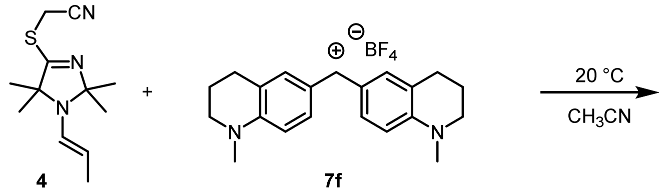

4 **7f**

[**4**][a] (M)	[**7f**] (M)	k_{obs} (s^{-1})
1.15×10^{-3}		9.00×10^{-3}
2.29×10^{-3}	4.98×10^{-5}	1.78×10^{-2}
3.43×10^{-3}		2.77×10^{-2}
4.58×10^{-3}		3.52×10^{-2}

$k_2 = 7.74\ M^{-1}\ s^{-1}$

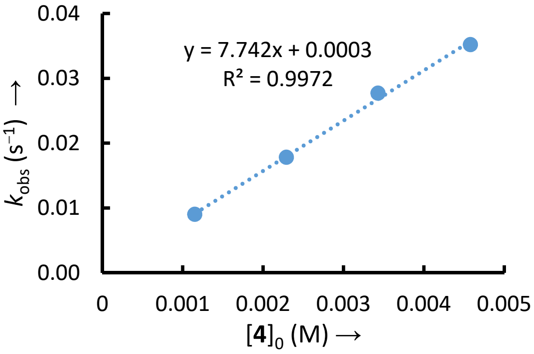

[a] The content of **4** in a mixture containing 2,6-lutidine, **3**, **4**, and acetonitrile (total: 99.3 mg) was determined to be 14.2 mg (see Figure S3 for the ^1H NMR spectrum of the sample). This mass was used to calculate the concentrations of the enamine **4.**

Table S6. Kinetics of the reactions of **4** with **7g** in CH_3CN at 20 °C (conventional UV/Vis method for [**4**] = 5.64×10^{-4} M, stopped-flow method for all other entries, λ = 614 nm)

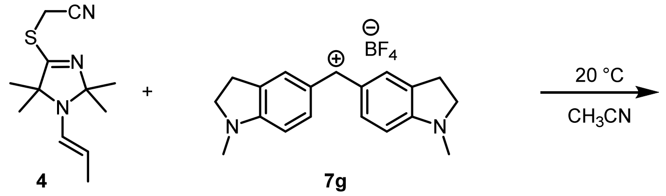

4 **7g**

[**4**][a] (M)	[**7g**] (M)	k_{obs} (s^{-1})
5.64×10^{-4}	1.53×10^{-5}	2.91×10^{-3}
1.83×10^{-3}		5.54×10^{-3}
2.81×10^{-3}	2.46×10^{-5}	7.98×10^{-3}
3.78×10^{-3}		1.07×10^{-2}

$k_2 = 2.42\ M^{-1}\ s^{-1}$

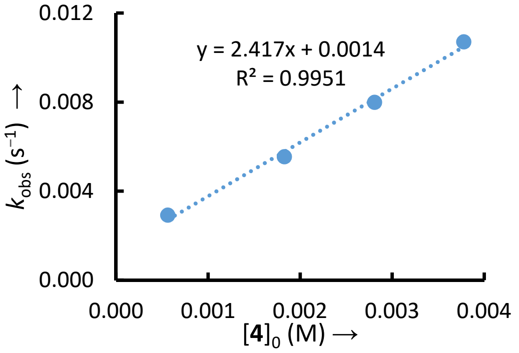

[a] The content of **4** in a mixture containing 2,6-lutidine, **3**, **4**, and acetonitrile (total: 226.3 mg) was determined to be 57.9 mg (see Figure S4 for the ^1H NMR spectrum of the sample). This mass was used to calculate the concentrations of the enamine **4.**

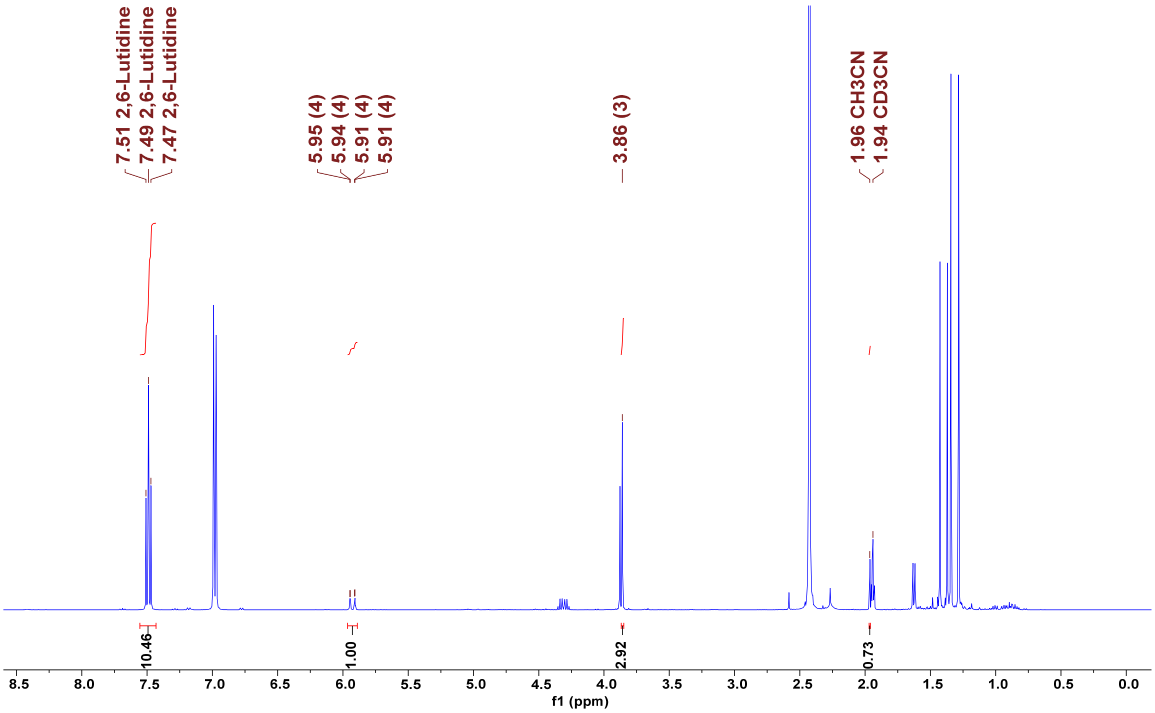

Figure S3. ^{1}H NMR spectrum (400 MHz, CD_3CN) with integrated and labelled resonances used to calculate the content of **4** present in the sample. This sample (MJH-I-183) was used to prepare the stock solutions of **4** for the experiments to determine the kinetics of the reactions of enamine **4** with **7d**, **7e**, and **7f**.

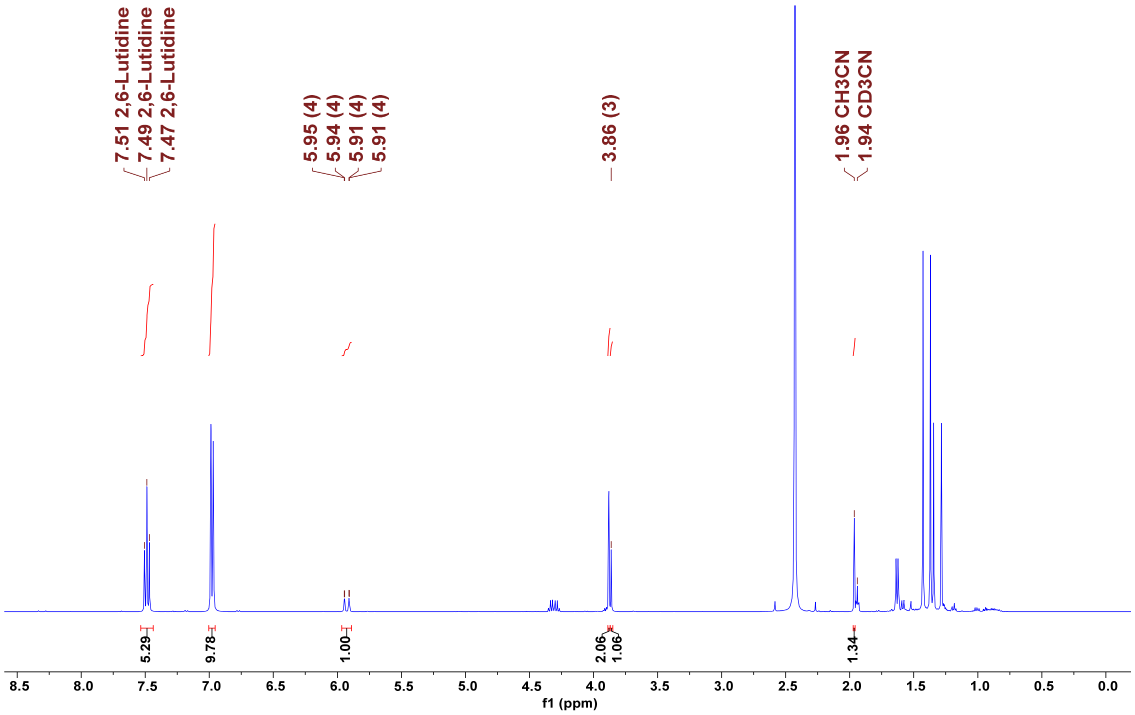

Figure S4. ^{1}H NMR spectrum (400 MHz, CD_3CN) with integrated and labelled resonances used to calculate the content of **4** present in the sample. This sample (MJH-I-124) was used to prepare the stock solutions of **4** for the experiments to determine the kinetics of the reactions of enamine **4** with **7g**.

9.2 Kinetics of the cinnamaldehyde-derived enamine 5 in acetonitrile

Table S7. Kinetics of the reactions of **5** with **7a** in CH_3CN at 20 °C (stopped flow, λ = 434 nm)

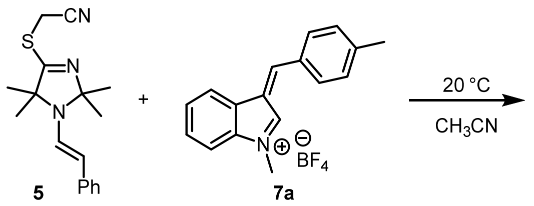

[**5**] (M)	[**7a**] (M)	k_{obs} (s^{-1})
4.97×10^{-4}		2.31×10^{1}
1.00×10^{-3}	4.87×10^{-5}	5.34×10^{1}
1.50×10^{-3}		8.54×10^{1}
2.00×10^{-3}		1.28×10^{2}

$k_2 = 6.92 \times 10^4\ M^{-1}\ s^{-1}$

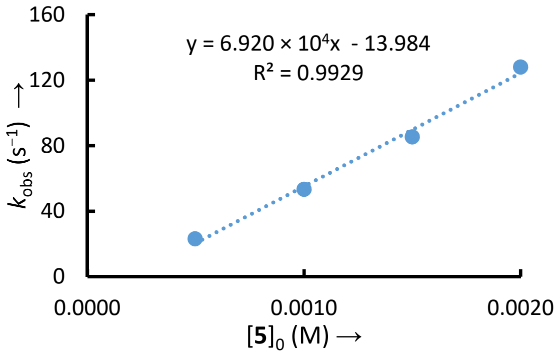

Table S8. Kinetics of the reactions of **5** with **7b** in CH_3CN at 20 °C (stopped-flow, λ = 586 nm)

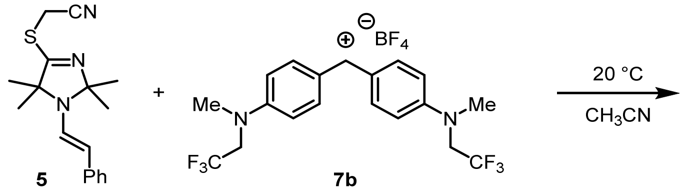

[**5**] (M)	[**7b**] (M)	k_{obs} (s^{-1})
5.12×10^{-4}		9.62×10^{-1}
1.02×10^{-3}	5.02×10^{-5}	1.94
1.53×10^{-3}		2.98
2.05×10^{-3}		3.97

$k_2 = 1.96 \times 10^3\ M^{-1}\ s^{-1}$

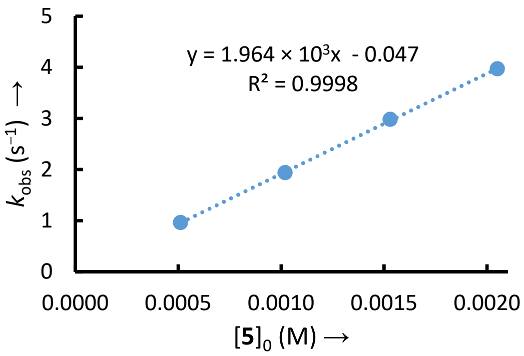

Table S9. Kinetics of the reactions of **5** with **7c** in CH_3CN at 20 °C (stopped-flow, λ = 612 nm)

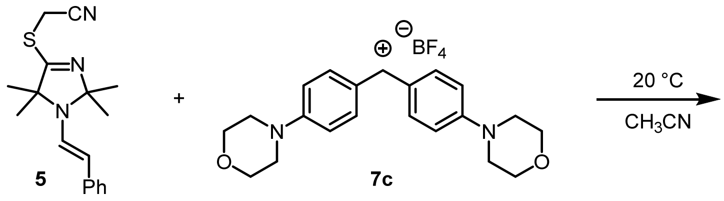

[**5**] (M)	[**7c**] (M)	k_{obs} (s^{-1})
5.12×10^{-4}		4.82×10^{-2}
1.02×10^{-3}	4.99×10^{-5}	9.26×10^{-2}
1.53×10^{-3}		1.38×10^{-1}
2.05×10^{-3}		1.84×10^{-1}

$k_2 = 8.84 \times 10^1$ M^{-1} s^{-1}

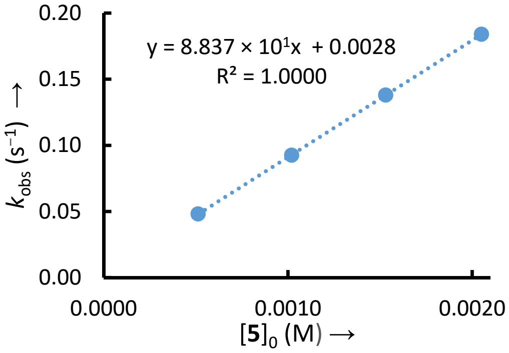

9.3 Correlations of lg k_2 for reactions of **7** with **4** and **5** in acetonitrile with the electrophilicities *E* of **7**

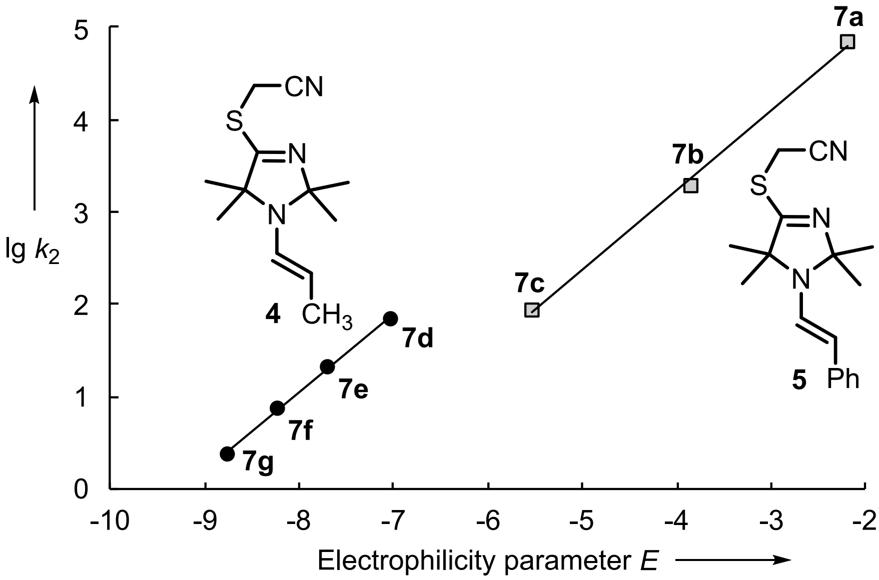

Figure S5. Linear relationships of lg k_2 for the reactions **4** + **7d-g** and **5** + **7a-c** (MeCN, 20 °C) with the electrophilicities *E* of benzhydrylium ions **7** (with data from Sections 9.1 and 9.2).

9.4 Kinetics of the cinnamaldehyde-derived enamine 5 in dichloromethane

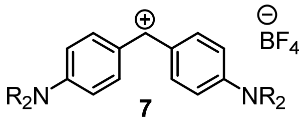

NR_2 = $N(Me)CH_2CF_3$ (**7b**), *E* = –3.85
$N(CH_2CH_2)_2O$ (**7c**), *E* = –5.53

Electrophiles used only in CH_2Cl_2
$N(Ph)CH_2CF_3$ (**7h**), *E* = –3.14
NPh_2 (**7i**), *E* = –4.72

Figure S6. Reference electrophiles **7** used for kinetic measurements in CH_2Cl_2.

Table S10. Kinetics of the reactions of **5** with **7h** in CH_2Cl_2 at 20 °C (stopped-flow, λ = 602 nm)

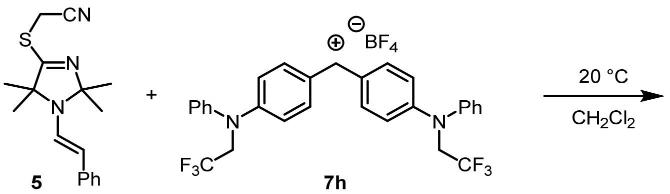

[**5**] (M)	[**7h**] (M)	k_{obs} (s^{-1})
5.16×10^{-4}		1.40×10^{1} [a]
1.03×10^{-3}	5.04×10^{-5}	3.03×10^{1} [a]
1.55×10^{-3}		4.66×10^{1} [a]
2.06×10^{-3}		6.78×10^{1} [a]
$k_2 = 3.45 \times 10^4\ M^{-1}\ s^{-1}$		

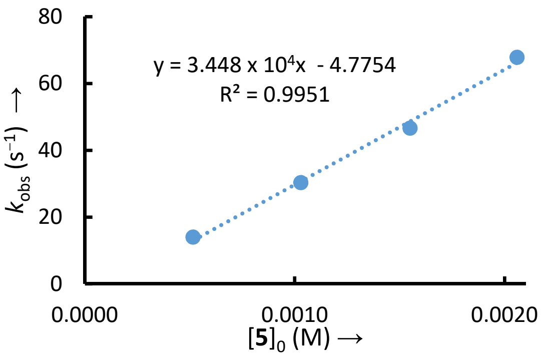

[a] Only the first half-life time was used to determined k_{obs}.

Table S11. Kinetics of the reactions of **5** with **7b** in CH_2Cl_2 at 20 °C (stopped-flow, λ = 594 nm)

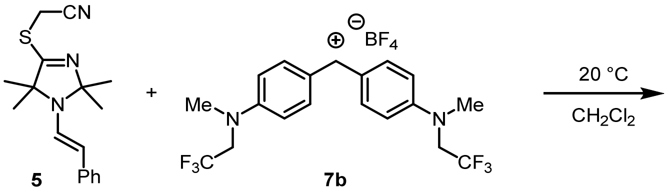

[**5**] (M)	[**7b**] (M)	k_{obs} (s^{-1})
5.16×10^{-4}		1.01
1.03×10^{-3}	5.08×10^{-5}	1.86
1.55×10^{-3}		2.77
2.06×10^{-3}		3.72

$k_2 = 1.75 \times 10^3$ M^{-1} s^{-1}

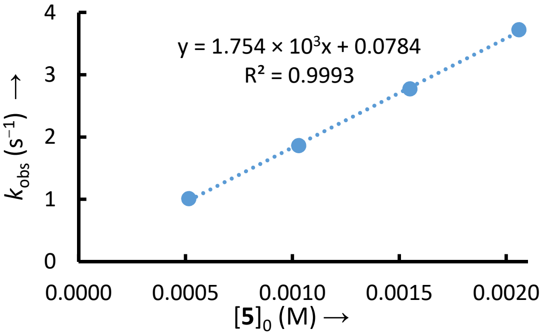

Table S12. Kinetics of the reactions of **5** with **7i** in CH_2Cl_2 at 20 °C (stopped-flow, λ = 674 nm)

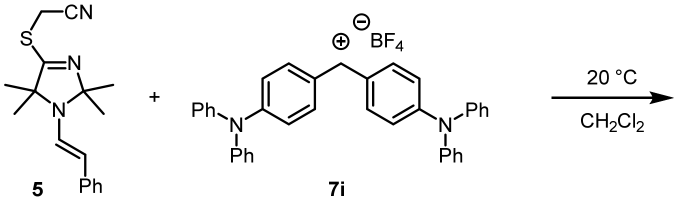

[**5**] (M)	[**7i**] (M)	k_{obs} (s^{-1})
5.16×10^{-4}		2.81×10^{-1}
1.03×10^{-3}	5.05×10^{-5}	5.06×10^{-1}
1.55×10^{-3}		7.53×10^{-1}
2.06×10^{-3}		1.00

$k_2 = 4.67 \times 10^2$ M^{-1} s^{-1}

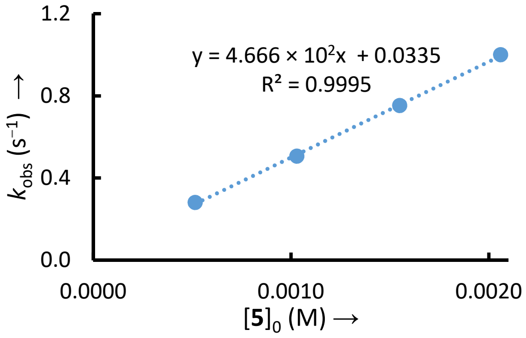

Table S13. Kinetics of the reactions of **5** with **7c** in CH_2Cl_2 at 20 °C (stopped-flow, λ = 622 nm)

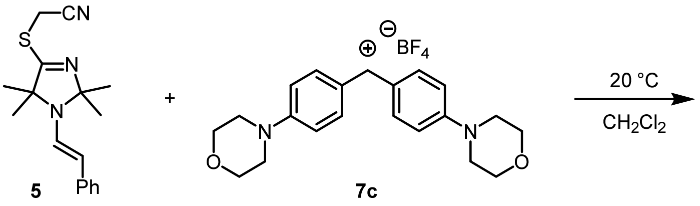

[**5**] (M)	[**7c**] (M)	k_{obs} (s^{-1})
5.16×10^{-4}	4.97×10^{-5}	6.90×10^{-2}
1.03×10^{-3}		9.79×10^{-2}
1.55×10^{-3}		1.26×10^{-1}
2.06×10^{-3}		1.54×10^{-1}

$k_2 = 5.49 \times 10^{1}\ M^{-1}\ s^{-1}$

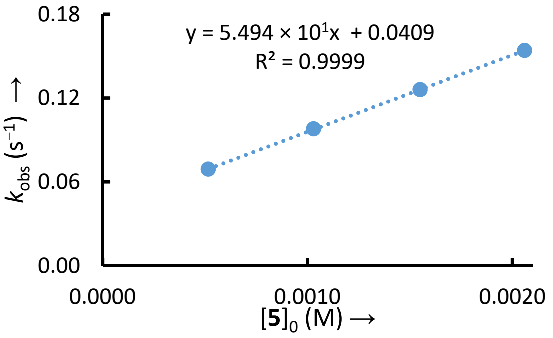

Table S14. Second-order rate constants k_2 for the reactions of the enamine **5** with benzhydrylium tetrafluoroborates **7** (CH_2Cl_2, 20 °C) and determination of the reactivity parameters N (and s_N) for **5** in dichloromethane.

Enamine	Electrophiles	Electrophilicity E	k_2 ($M^{-1}\ s^{-1}$)	N, s_N
**5**	**7h**	−3.14	3.45×10^{4}	7.06, 1.11
	**7b**	−3.85	1.75×10^{3}	
	**7i**	−4.72	4.67×10^{2}	
	**7c**	−5.53	5.49×10^{1}	

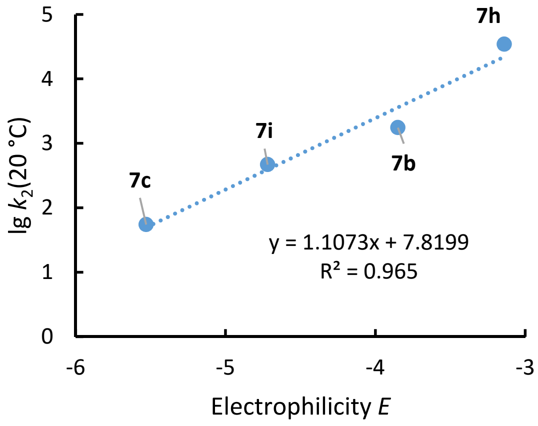

10. Copies of ^{1}H NMR, ^{13}C NMR, and IR spectra

2-((2,2,5,5-Tetramethyl-2,5-dihydro-1*H*-imidazol-4-yl)thio)acetonitrile (3)

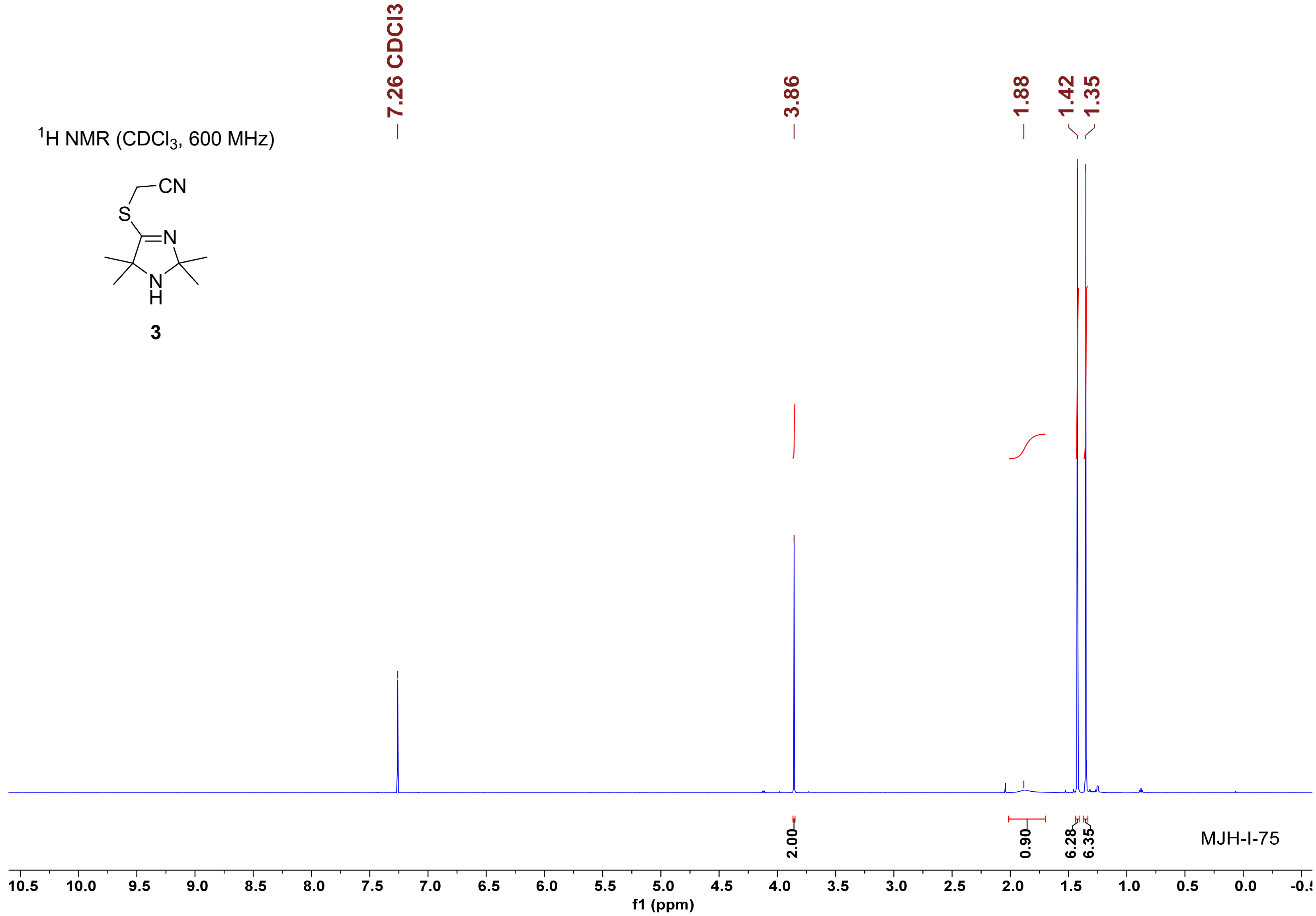

2-((2,2,5,5-Tetramethyl-2,5-dihydro-1*H*-imidazol-4-yl)thio)acetonitrile (3)

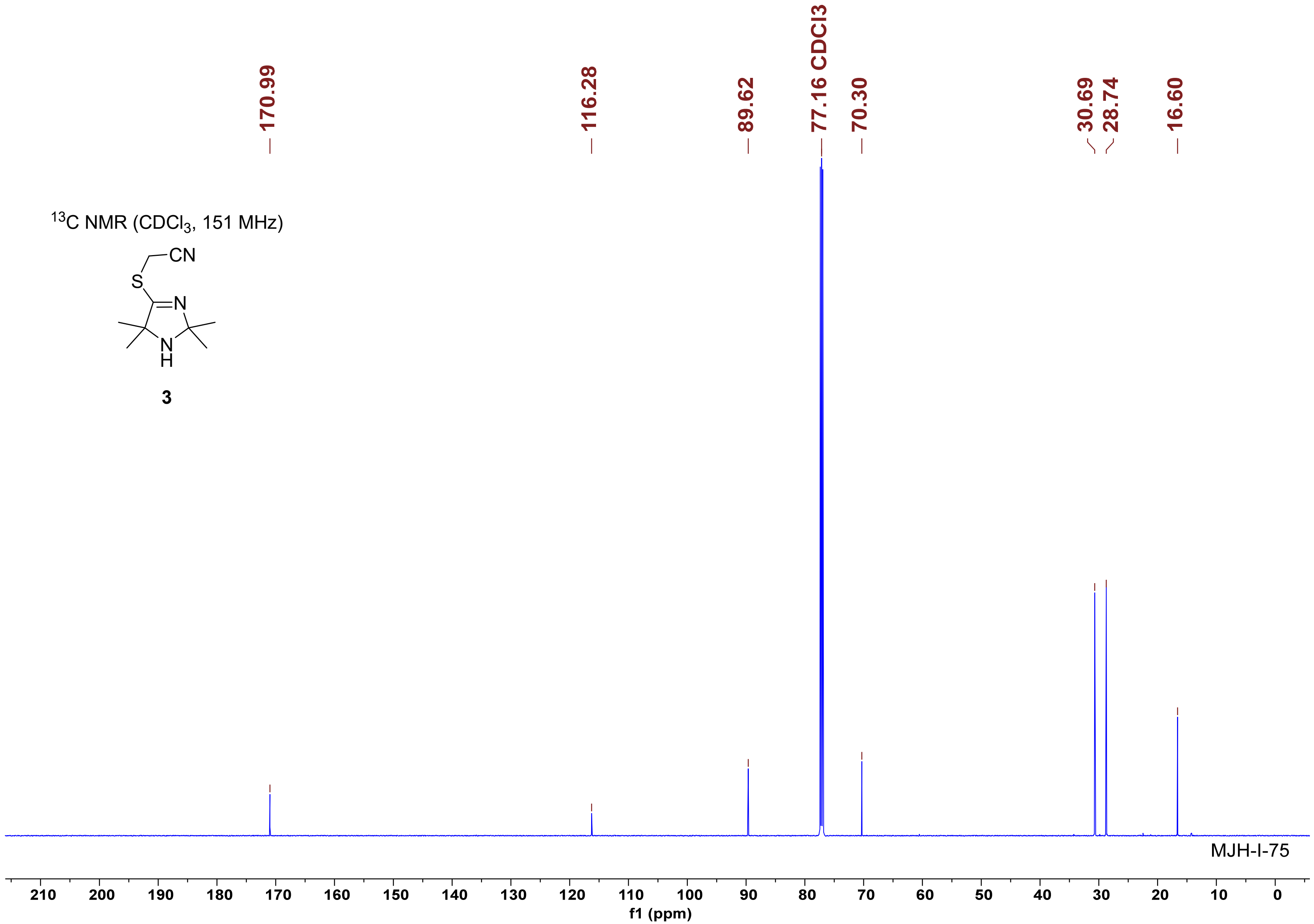

2-((2,2,5,5-Tetramethyl-2,5-dihydro-1*H*-imidazol-4-yl)thio)acetonitrile (3)

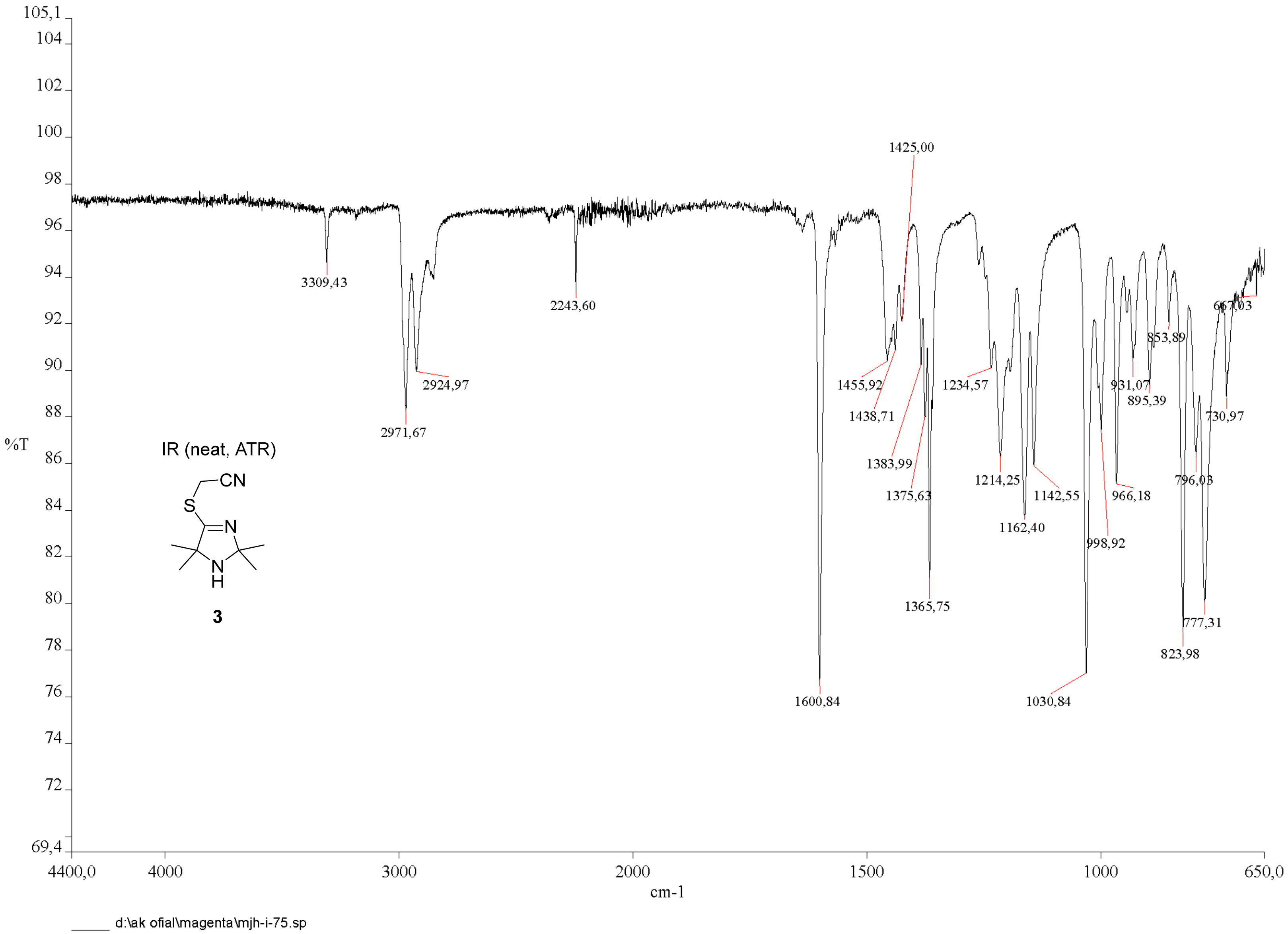

(*E*)-2-((2,2,5,5-Tetramethyl-1-(prop-1-en-1-yl)-2,5-dihydro-1*H*-imidazol-4-yl)thio)acetonitrile (4)

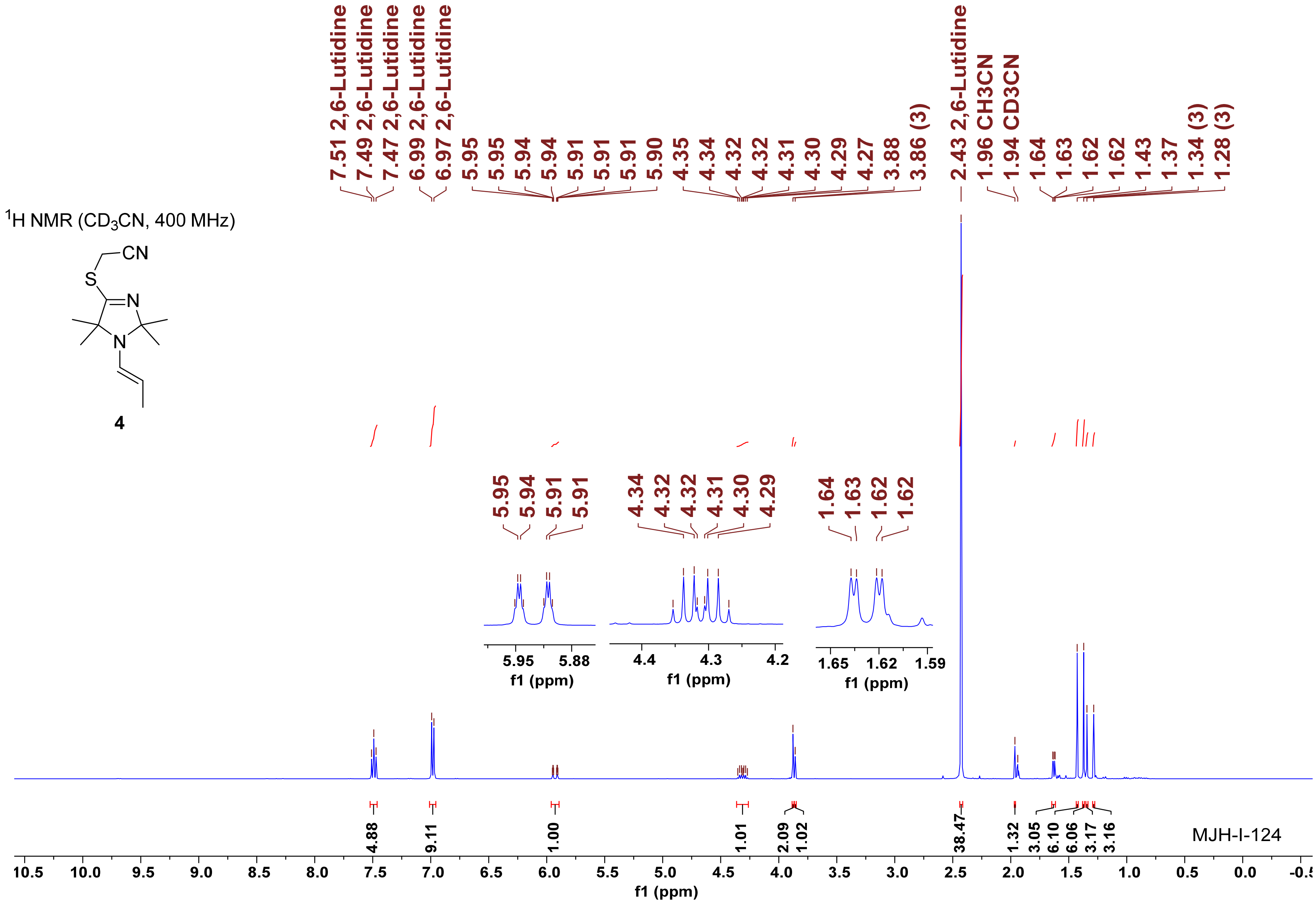

(*E*)-2-((2,2,5,5-Tetramethyl-1-(prop-1-en-1-yl)-2,5-dihydro-1*H*-imidazol-4-yl)thio)acetonitrile (4)

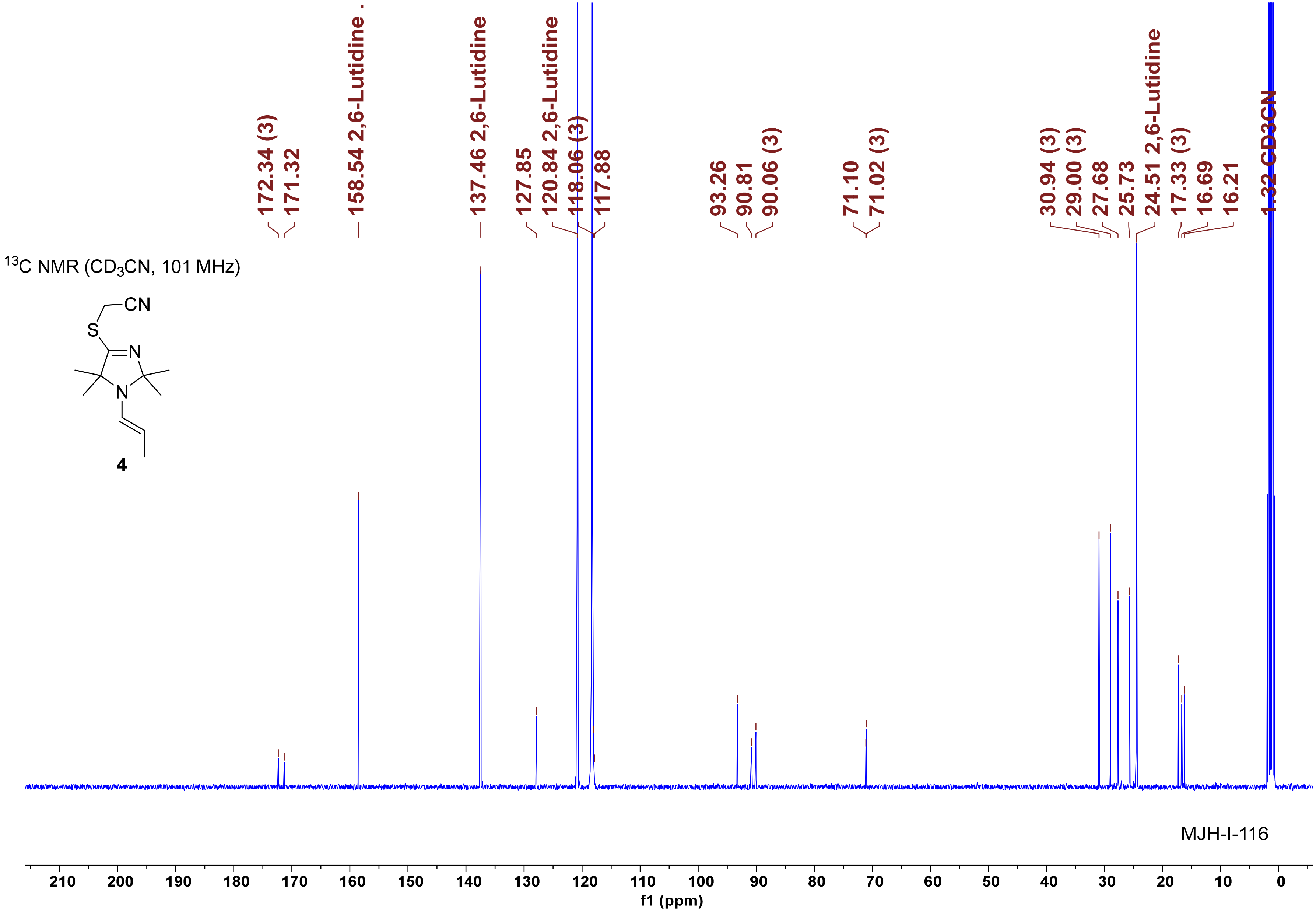

(*E*)-2-((2,2,5,5-Tetramethyl-1-styryl-2,5-dihydro-1*H*-imidazol-4-yl)thio)acetonitrile (5)

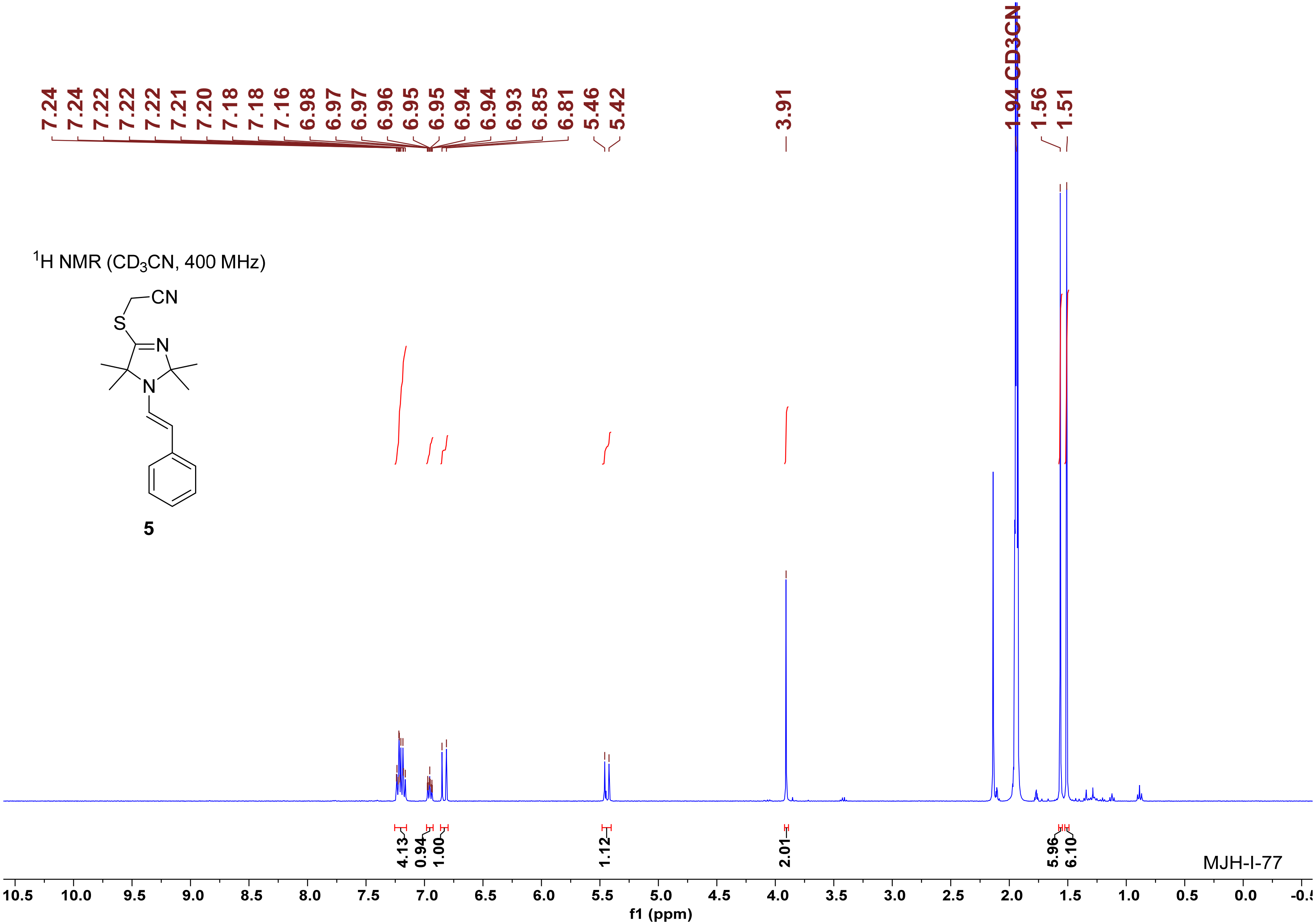

(*E*)-2-((2,2,5,5-Tetramethyl-1-styryl-2,5-dihydro-1*H*-imidazol-4-yl)thio)acetonitrile (5)

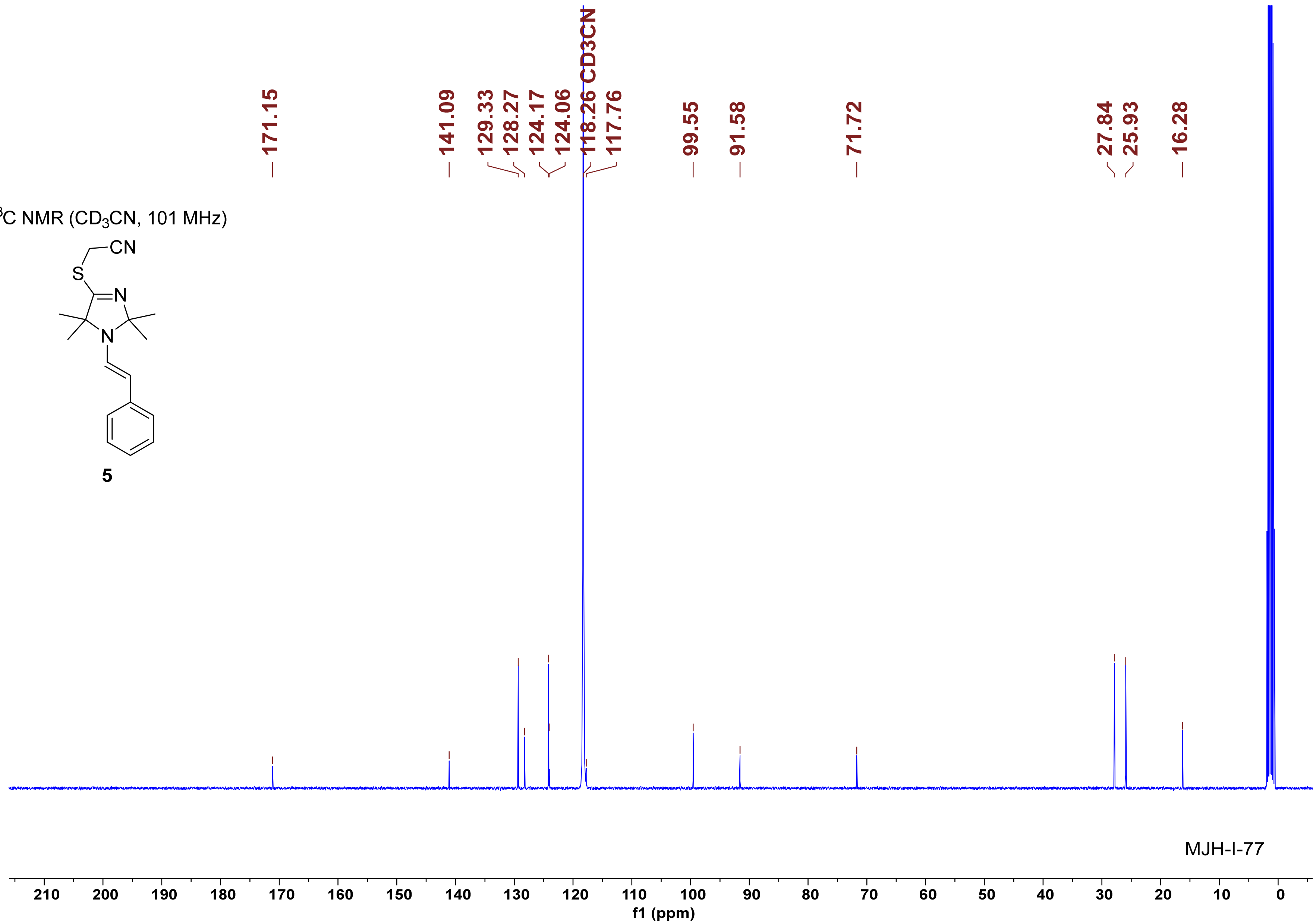

5

(*E*)-2-((2,2,5,5-Tetramethyl-1-styryl-2,5-dihydro-1*H*-imidazol-4-yl)thio)acetonitrile (5)

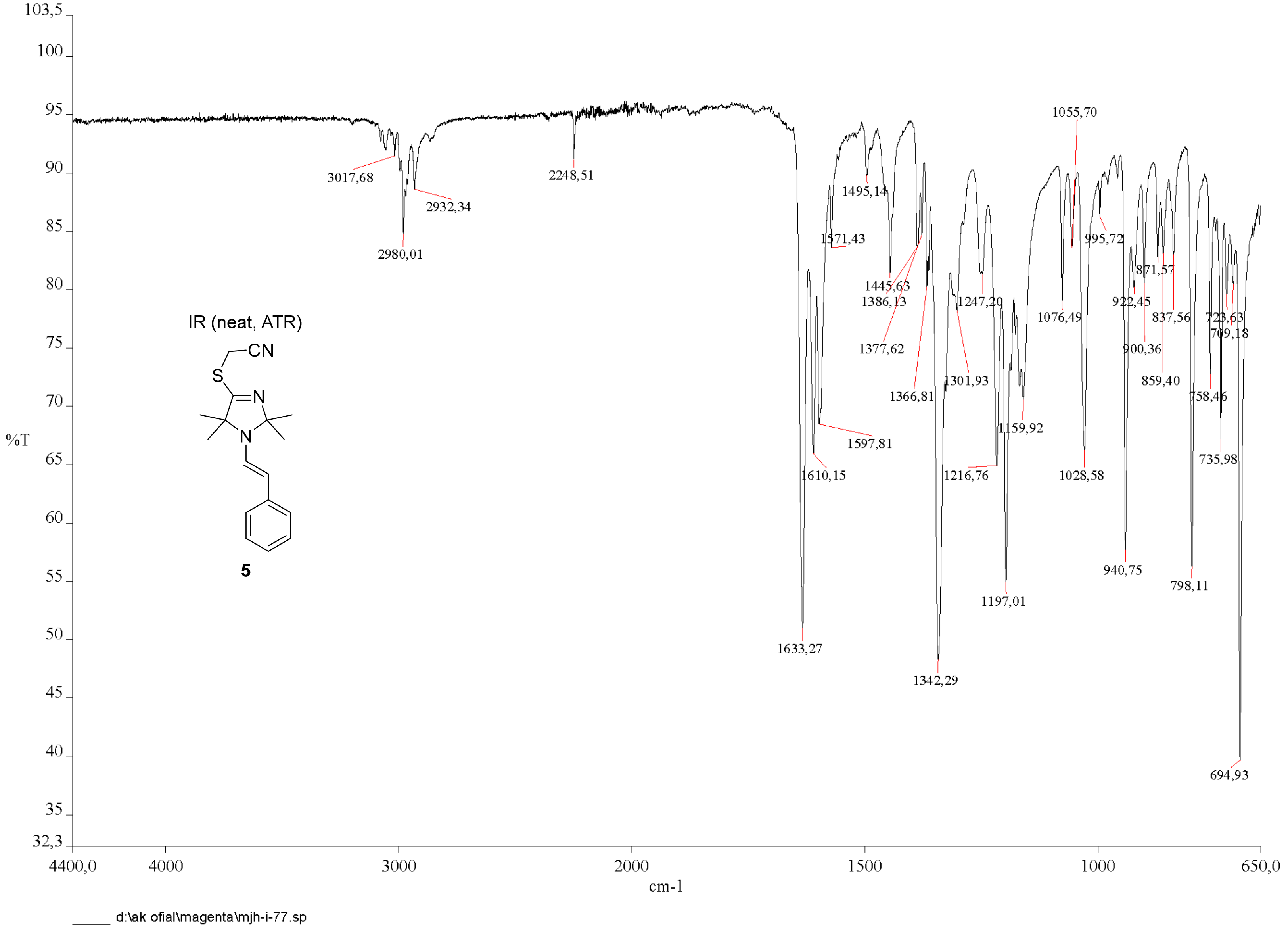

3,3-Bis(4-(dimethylamino)phenyl)-2-methylpropanal (9a)

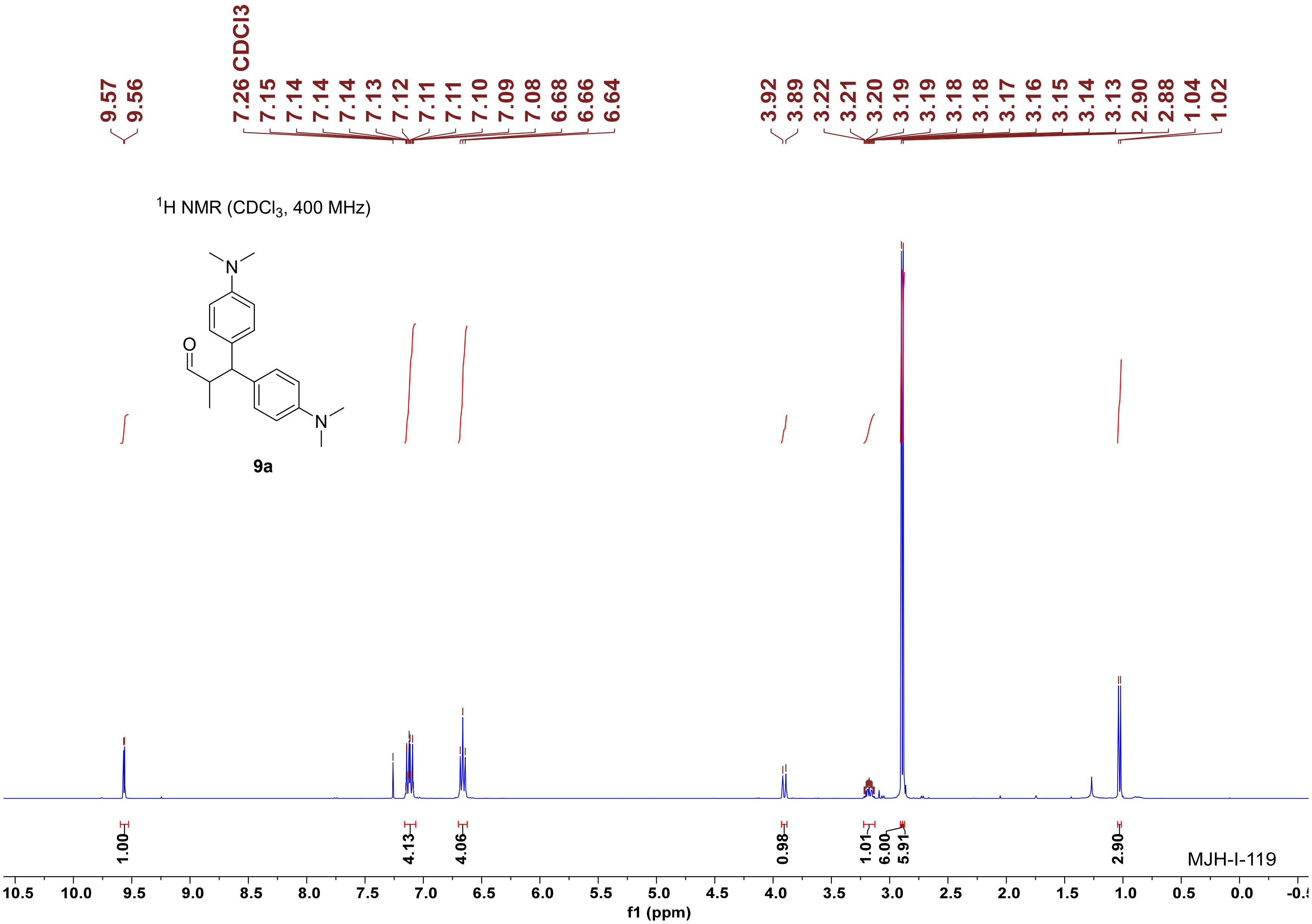

3,3-Bis(4-(dimethylamino)phenyl)-2-methylpropanal (9a)

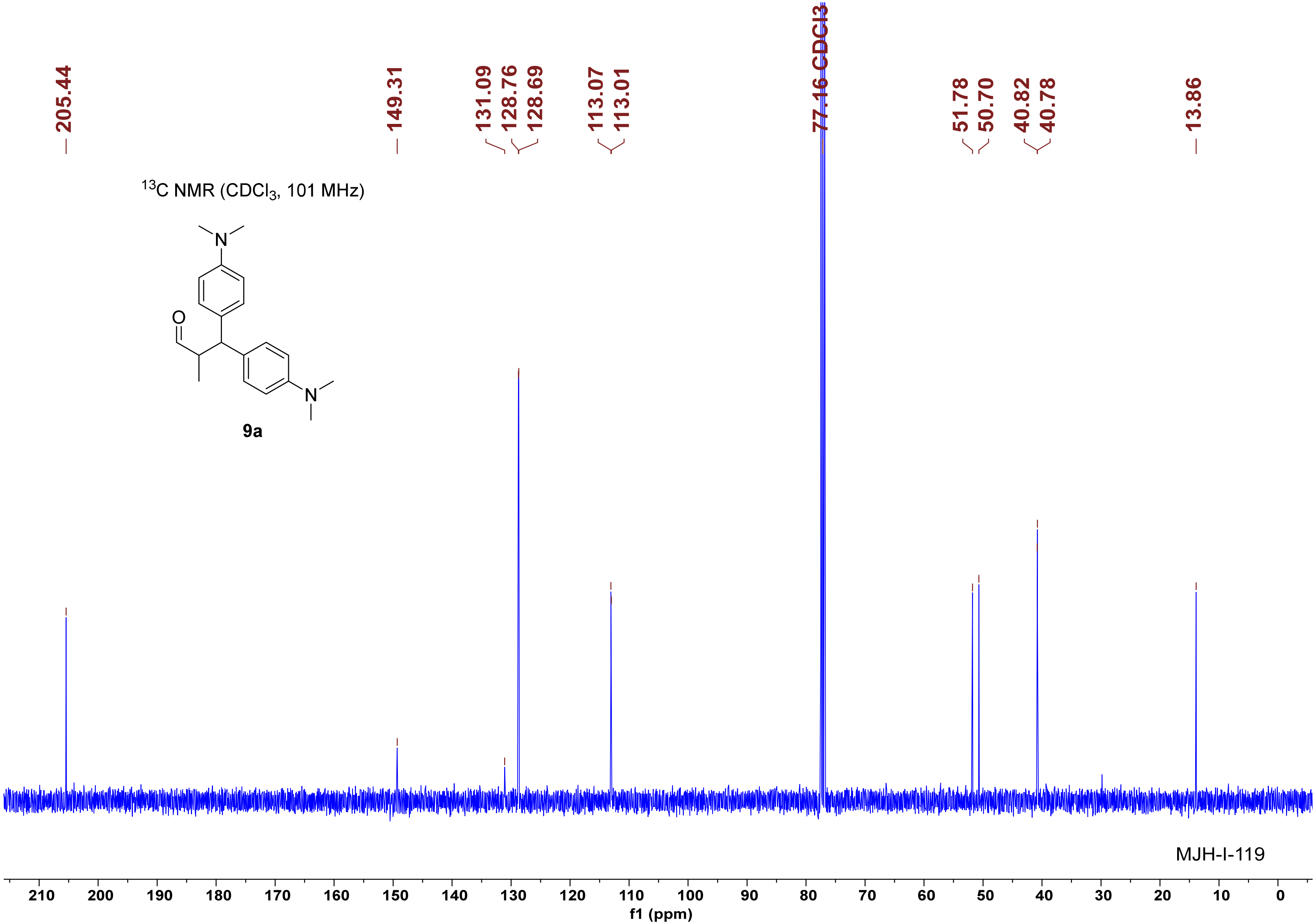

2-Methyl-3,3-bis(4-(pyrrolidin-1-yl)phenyl)propanal (9b)

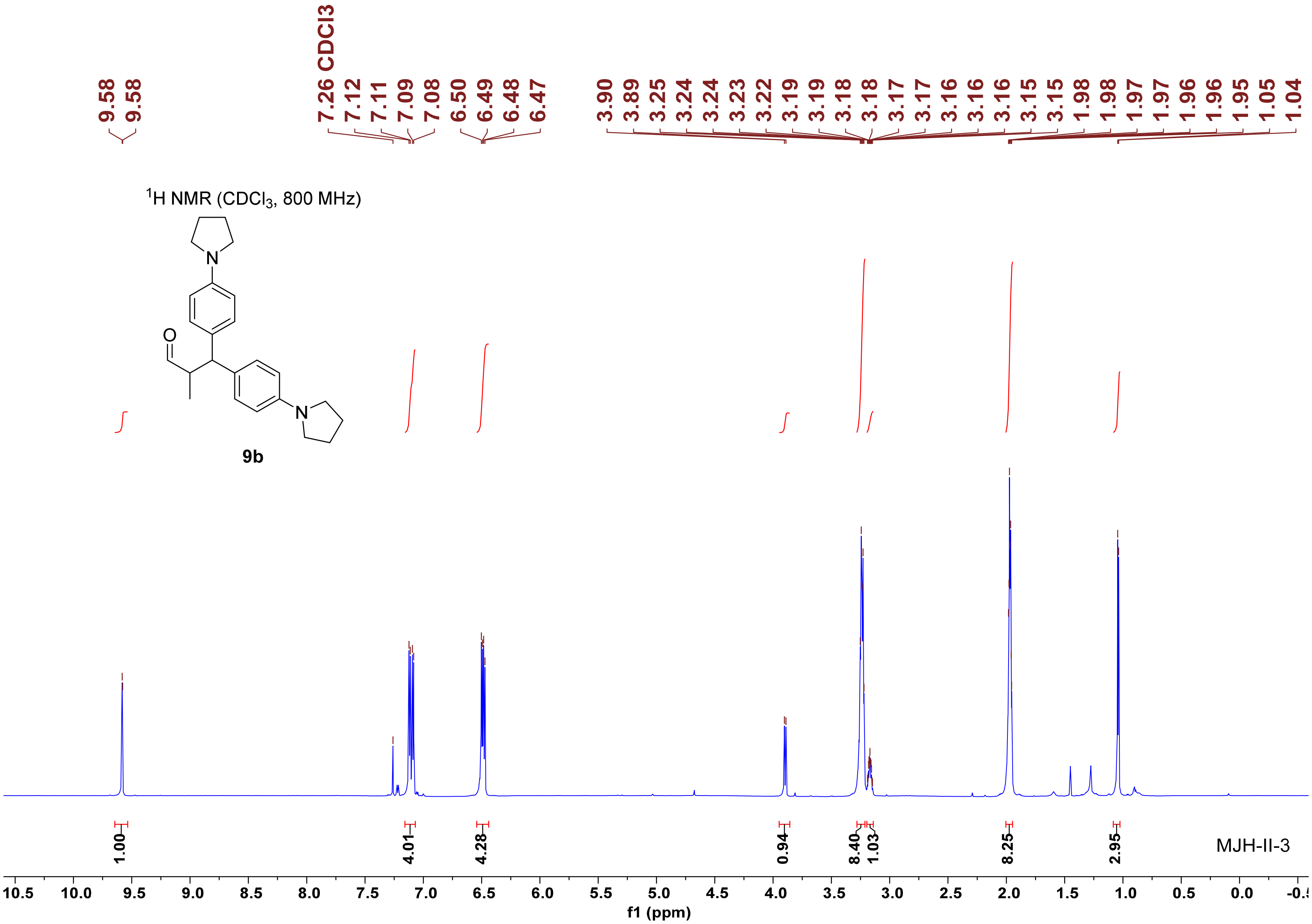

2-Methyl-3,3-bis(4-(pyrrolidin-1-yl)phenyl)propanal (9b)

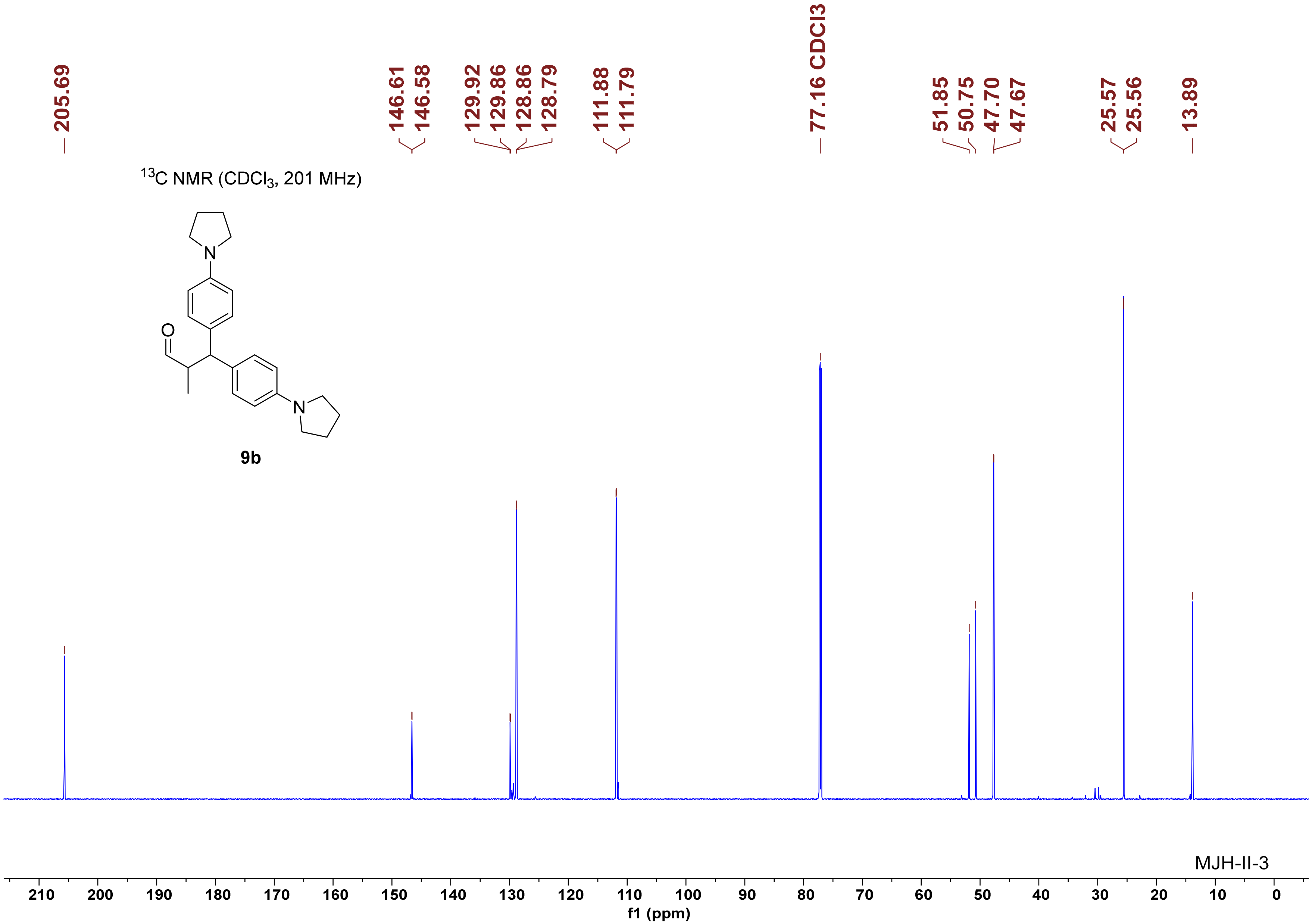

2-Methyl-3,3-bis(4-(pyrrolidin-1-yl)phenyl)propanal (9b)

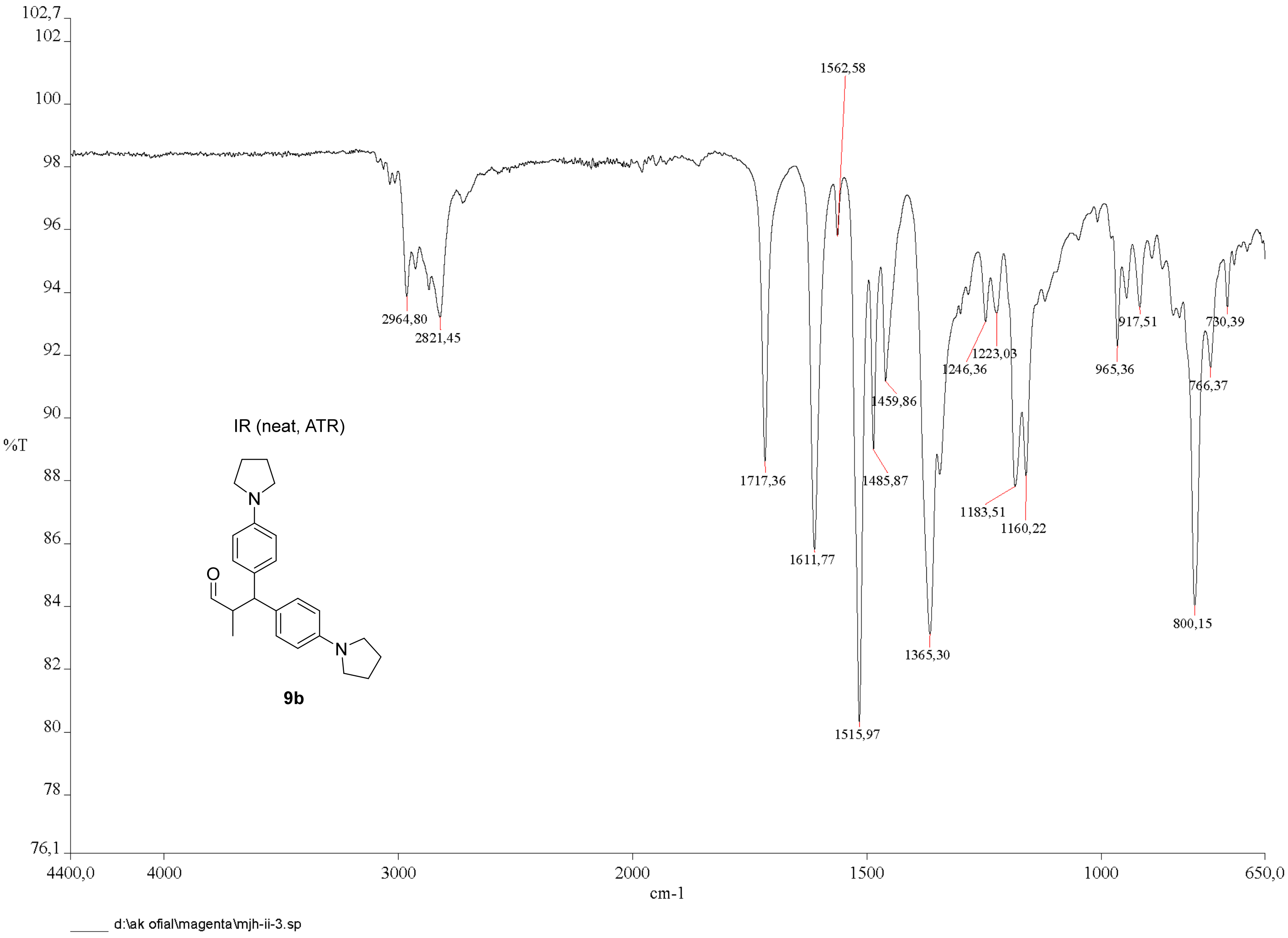

2-Methyl-3,3-bis(1-methylindolin-5-yl)propanal (9c)

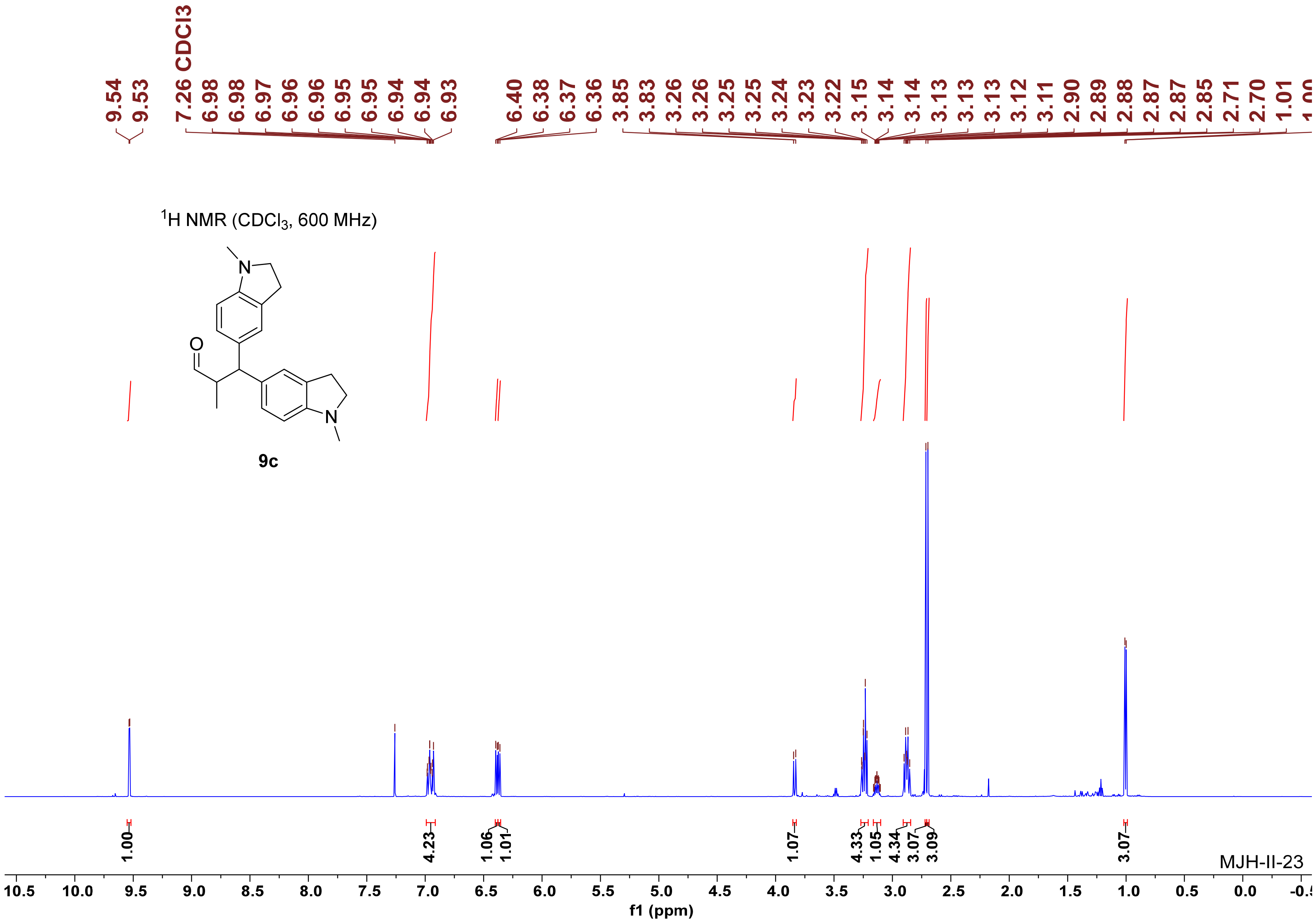

2-Methyl-3,3-bis(1-methylindolin-5-yl)propanal (9c)

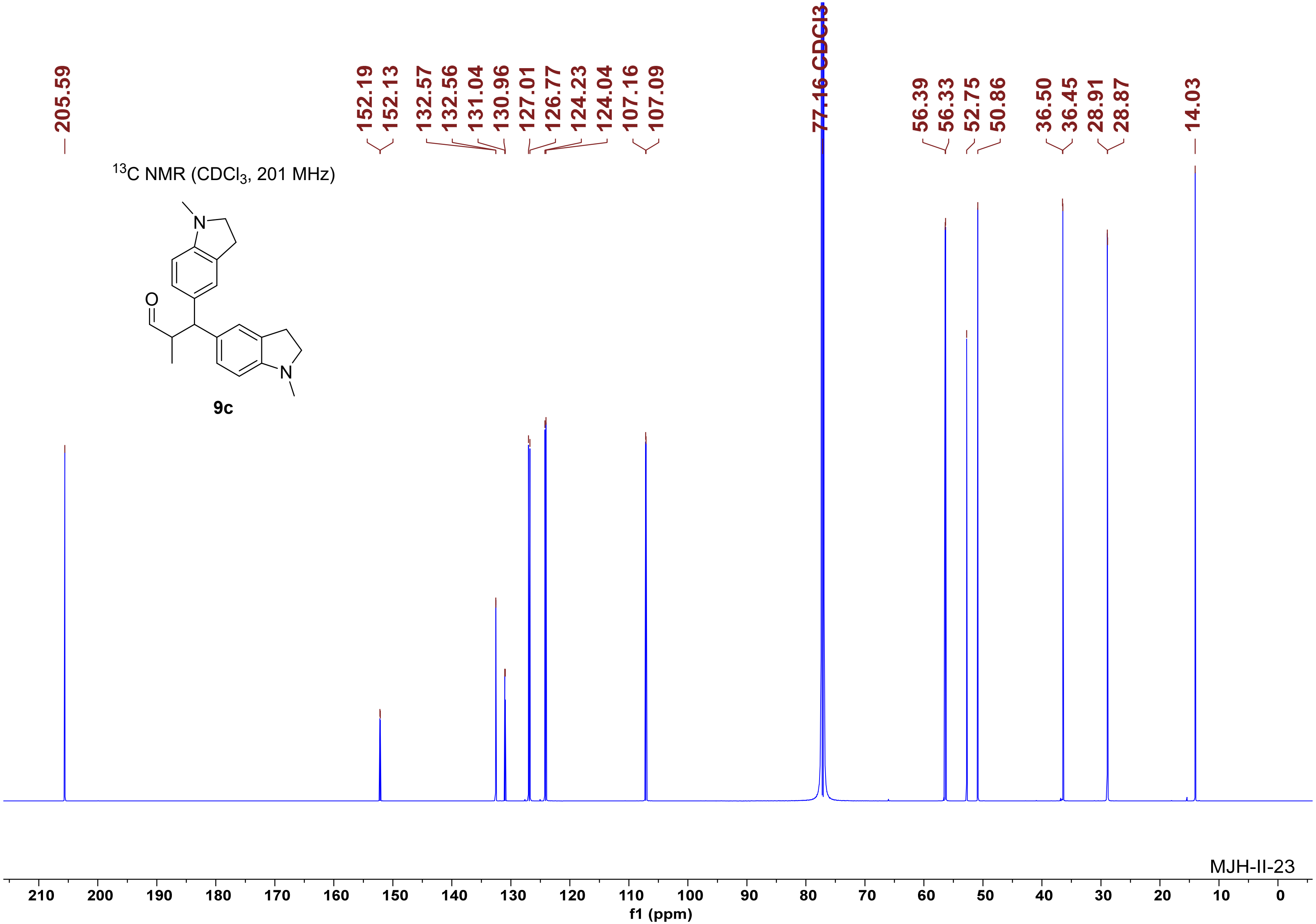

2-Methyl-3,3-bis(1-methylindolin-5-yl)propanal (9c)

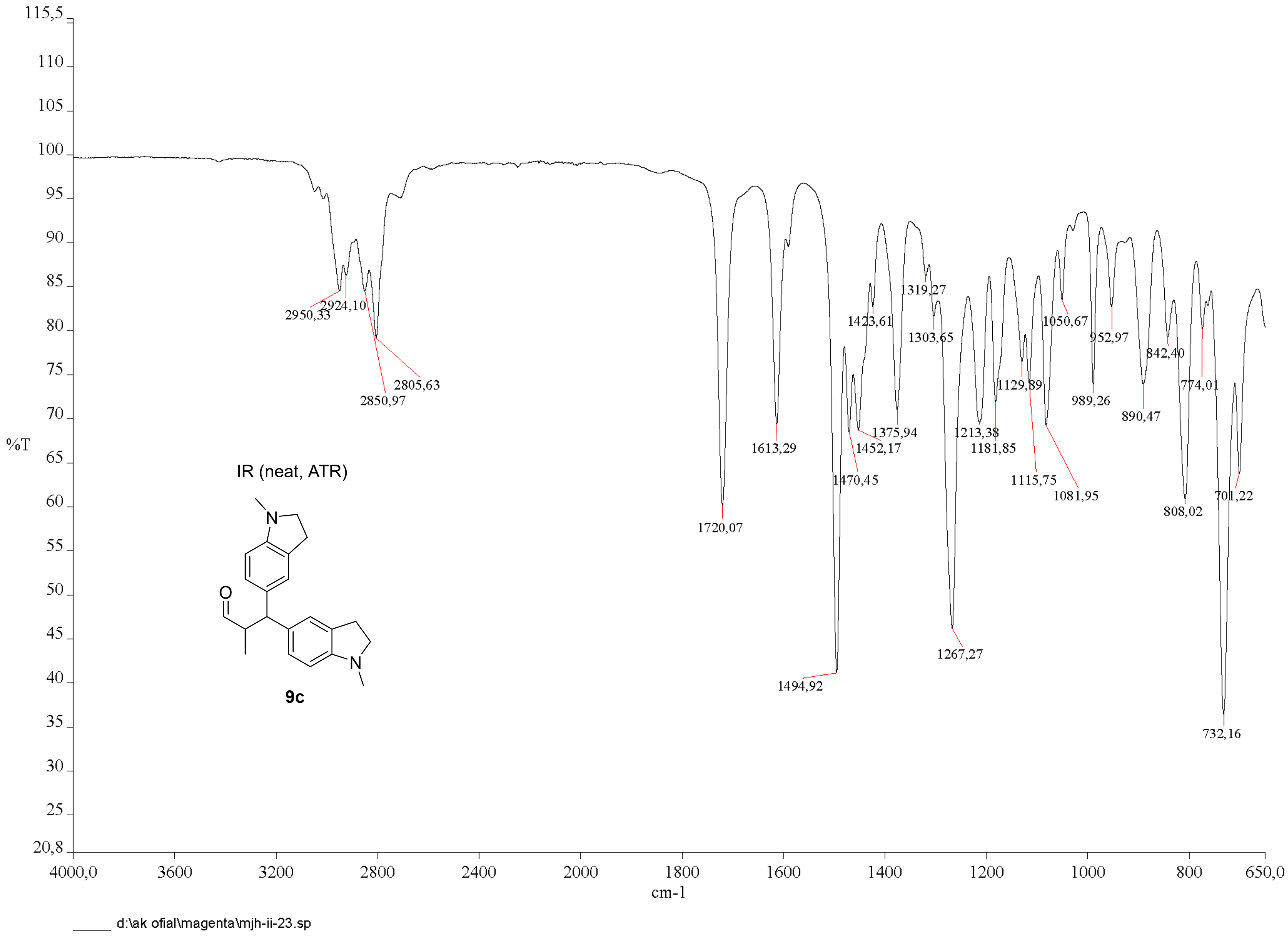

3,3-Bis(4-morpholinophenyl)-2-phenylpropanal (9d)

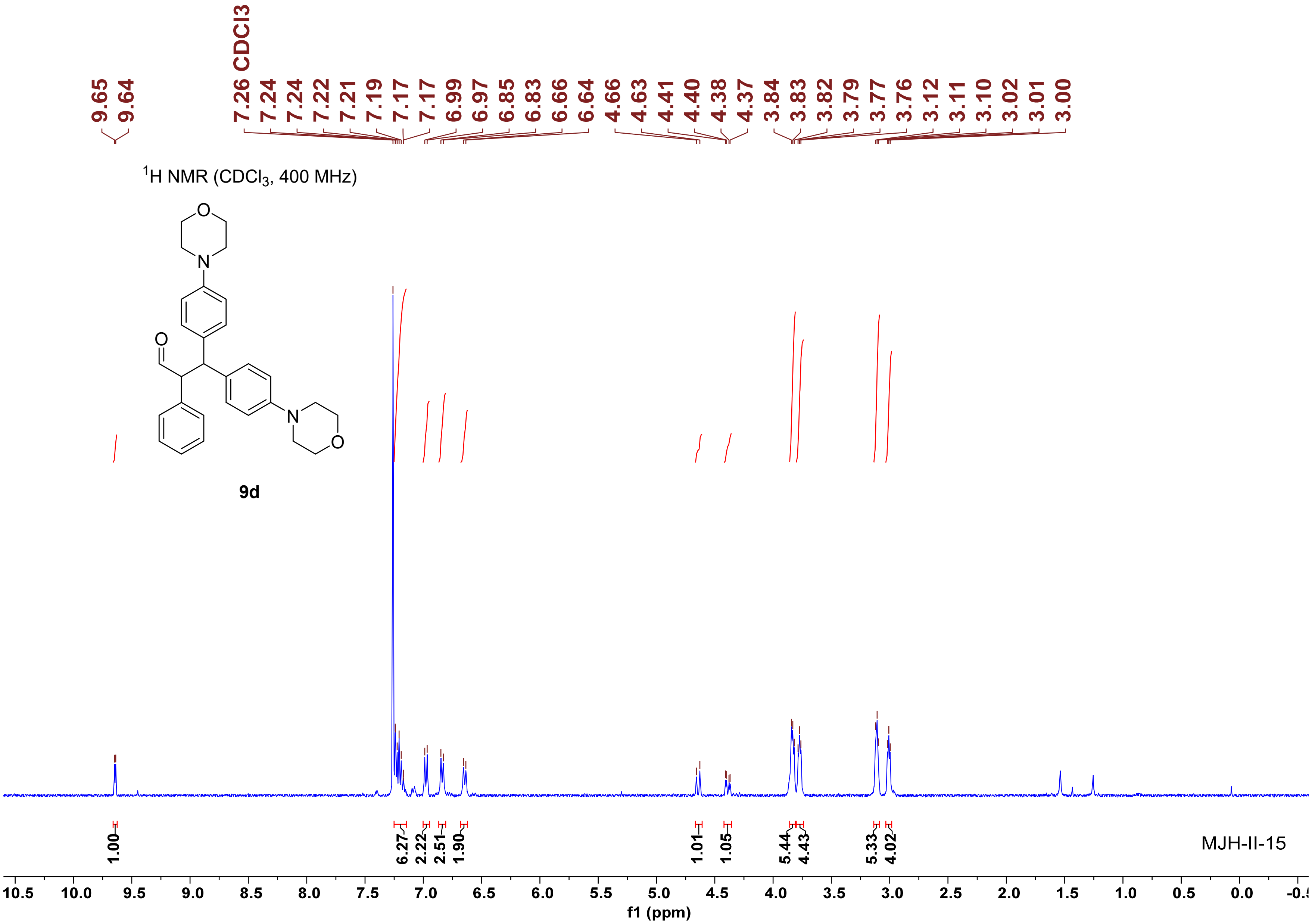

3,3-Bis(4-morpholinophenyl)-2-phenylpropanal (9d)

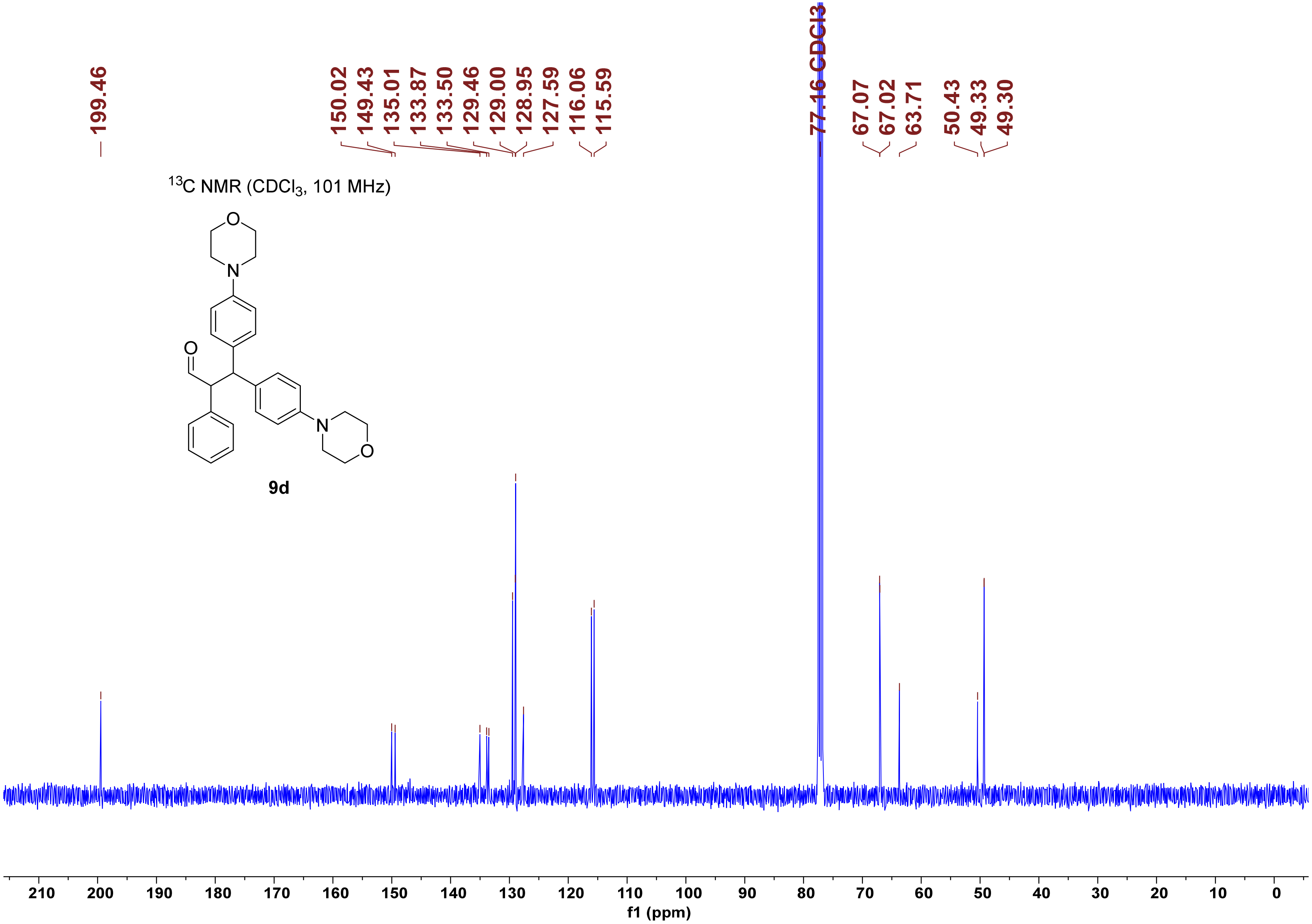

3,3-Bis(4-morpholinophenyl)-2-phenylpropanal (9d)

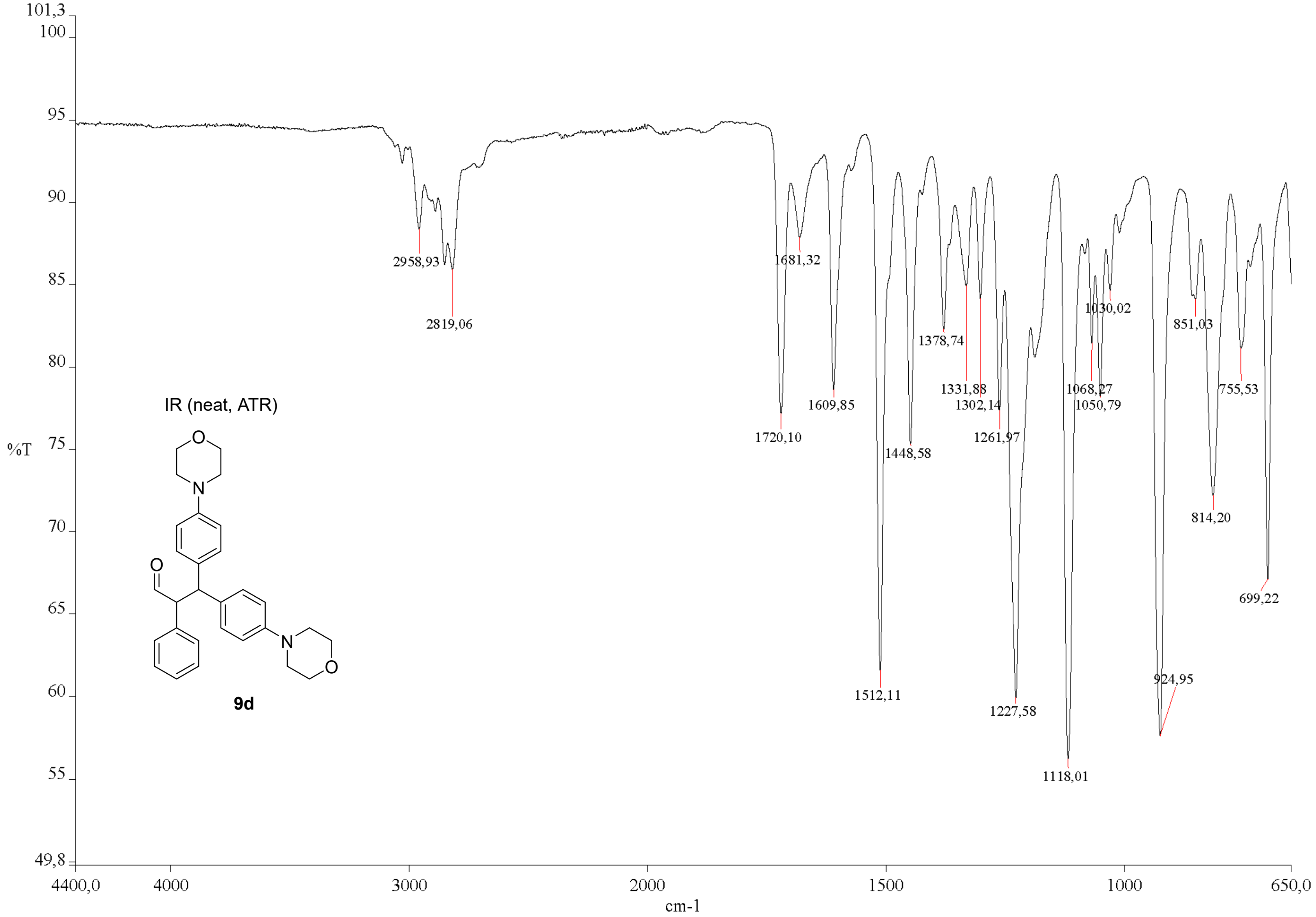

11. References

(S1) a) J. D. Christian, *J. Org. Chem.*, 1957, **22**, 396-399. b) A. C. Closs, E. Fuks, M. Bechtel, O. Trapp, *Chem. Eur. J.*, 2020, **26**, 10702-10706.

(S2) a) E. Follet, G. Berionni, P. Mayer, H. Mayr, *J. Org. Chem.*, 2015, **80**, 8643-8656. b) R. J. Mayer, N. Hampel, P. Mayer, A. R. Ofial, H. Mayr, *Eur. J. Org. Chem.*, 2019*,* **2019**, 412-421.

(S3) G. R. Fulmer, A. J. M. Miller, N. H. Sherden, H. E. Gottlieb, A. Nudelman, B. M. Stolz, J. E. Bercaw, K. I. Goldberg, *Organometallics*, 2010, **29**, 2176-2179.

(S4) S. Lakhdar, B. Maji, H. Mayr, *Angew. Chem. Int. Ed.*, 2012, **51**, 5739.

(S5) Bruker AXS Inc., *SAINT*, Madison, Wisconsin, USA, 2012.

(S6) G. M. Sheldrick, *SADABS*, University of Göttingen, Germany, 1996.

(S7) G. M. Sheldrick, *Acta. Cryst.*, 2015, **A71**, 3-8.

(S8) L. J. Farrugia, *J. Appl. Cryst.*, 2012, **45**, 849-854.

(S9) P. G. Cozzi, F. Benfatti, L. Zoli, *Angew. Chem. Int. Ed.*, 2009, **48**, 1313-1316.

(S10) a) A. C. Closs, M. Bechtel, O. Trapp, *Angew. Chem. Int. Ed*., 2022, **61**, e202112563; see also b) R. G. Thorat, A. M. Harned, *Chem. Commun*., 2018, **54**, 214.
